# GPR68-ATF4 signaling is a novel prosurvival pathway in glioblastoma activated by acidic extracellular microenvironment

**DOI:** 10.1186/s40164-023-00468-1

**Published:** 2024-01-31

**Authors:** Charles H. Williams, Leif R. Neitzel, Jessica Cornell, Samantha Rea, Ian Mills, Maya S. Silver, Jovanni D. Ahmad, Konstantin G. Birukov, Anna Birukova, Henry Brem, Betty Tyler, Eli E. Bar, Charles C. Hong

**Affiliations:** 1 Department of Medicine, Michigan State University College of Human Medicine, East Lansing, MI USA; 2 Henry Ford Health + Michigan State Health Sciences, Detroit, MI USA; 3 Department of Medicine, University of Maryland School of Medicine, Baltimore, MD USA; 4 Department of Pathology, University of Maryland School of Medicine, Baltimore, MD USA; 5 Department of Anesthesiology, University of Maryland School of Medicine, Baltimore, MD USA; 6 Department of Neurosurgery, Johns Hopkins University School of Medicine, Baltimore, MD USA; 7 Department of Neurosurgery, University of Maryland School of Medicine, Baltimore, MD USA; 8 University of Maryland Marlene and Stewart Greenebaum Comprehensive Cancer Center, Baltimore, MD USA

## Abstract

**Background:**

Glioblastoma multiforme (GBM) stands as a formidable challenge in oncology because of its aggressive nature and severely limited treatment options. Despite decades of research, the survival rates for GBM remain effectively stagnant. A defining hallmark of GBM is a highly acidic tumor microenvironment, which is thought to activate pro-tumorigenic pathways. This acidification is the result of altered tumor metabolism favoring aerobic glycolysis, a phenomenon known as the Warburg effect. Low extracellular pH confers radioresistant tumors to glial cells. Notably GPR68, an acid sensing GPCR, is upregulated in radioresistant GBM. Usage of Lorazepam, which has off target agonism of GPR68, is linked to worse clinical outcomes for a variety of cancers. However, the role of tumor microenvironment acidification in GPR68 activation has not been assessed in cancer. Here we interrogate the role of GPR68 specifically in GBM cells using a novel highly specific small molecule inhibitor of GPR68 named Ogremorphin (OGM) to induce the iron mediated cell death pathway: ferroptosis.

**Method:**

OGM was identified in a non-biased zebrafish embryonic development screen and validated with Morpholino and CRISPR based approaches. Next, A GPI-anchored pH reporter, pHluorin2, was stably expressed in U87 glioblastoma cells to probe extracellular acidification. Cell survival assays, via nuclei counting and cell titer glo, were used to demonstrate sensitivity to GPR68 inhibition in twelve immortalized and PDX GBM lines. To determine GPR68 inhibition’s mechanism of cell death we use DAVID pathway analysis of RNAseq. Our major indication, ferroptosis, was then confirmed by western blotting and qRT-PCR of reporter genes including TFRC. This finding was further validated by transmission electron microscopy and liperfluo staining to assess lipid peroxidation. Lastly, we use siRNA and CRISPRi to demonstrate the critical role of ATF4 suppression via GPR68 for GBM survival.

**Results:**

We used a pHLourin2 probe to demonstrate how glioblastoma cells acidify their microenvironment to activate the commonly over expressed acid sensing GPCR, GPR68. Using our small molecule inhibitor OGM and genetic means, we show that blocking GPR68 signaling results in robust cell death in all thirteen glioblastoma cell lines tested, irrespective of genetic and phenotypic heterogeneity, or resistance to the mainstay GBM chemotherapeutic temozolomide. We use U87 and U138 glioblastoma cell lines to show how selective induction of ferroptosis occurs in an ATF4-dependent manner. Importantly, OGM was not-acutely toxic to zebrafish and its inhibitory effects were found to spare non-malignant neural cells.

**Conclusion:**

These results indicate GPR68 emerges as a critical sensor for an autocrine pro-tumorigenic signaling cascade triggered by extracellular acidification in glioblastoma cells. In this context, GPR68 suppresses ATF4, inhibition of GPR68 increases expression of ATF4 which leads to ferroptotic cell death. These findings provide a promising therapeutic approach to selectively induce ferroptosis in glioblastoma cells while sparing healthy neural tissue.

**Supplementary Information:**

The online version contains supplementary material available at 10.1186/s40164-023-00468-1.

## Introduction

Glioblastoma multiforme (GBM) stands as the most prevalent and lethal primary brain tumor among adults, with a grim median survival of only 14 months despite aggressive standard management strategies [1]. The current approach involves maximal surgical resection, followed by radiation and chemotherapy using the frontline agent temozolomide (TMZ). Unfortunately, therapeutic resistance and molecular heterogeneity contribute to the recurring nature of GBM, with nearly ubiquitous TMZ resistance attributed to the induction of the DNA repair enzyme O(6)-methylguanine-DNA methyltransferase, encoded by the MGMT gene [2–4]. Moreover, the high molecular heterogeneity of GBM tumors, exemplified by unevenly distributed amplifications of EGFR and PDGFR, challenges the effectiveness of receptor tyrosine kinase inhibitors. Analysis of The Cancer Genome Atlas (TCGA) and single-cell RNA-seq further reveals substantial heterogeneity among patients and within primary GBM tumors, suggesting a formidable obstacle to identifying a universal therapeutic target [5, 6].

Despite variations in cell states and genetic makeup, a distinctive feature of glioblastoma is its acidic extracellular tumor microenvironment (TME), a result of the Warburg effect, which is a hallmark contributing to cancer progression by fostering malignant clonal selection, metastasis, and immune escape [7–14]. Extracellular acidification induces pro-oncogenic transcriptional responses, providing a growth advantage to the tumor [15]. However, the mechanism through which cancer cells sense these extracellular pH changes remains unclear. In medulloblastoma cells, extracellular acidification triggers calcium (Ca^2+^) fluxes in a phospholipase C (PLC)-dependent manner, implicating the involvement of a GPCR [16]. GPR68, also known as ovarian cancer G-coupled protein receptor 1 (OGR-1), is a member of the proton sensing GPCR family which is activated in response to subtle extracellular acidification (inactive at pH 7.4 and fully active at pH 6.4) [17]. Moreover, low extracellular pH confers radio-resistance and GPR68 is upregulated in radioresistant cell lines [18, 19]. Mounting evidence implicates acid-sensing GPCRs in the progression of various cancers, [20] with prior literature suggesting that anti-tumor effects of GPR68 inhibition involves modulation of cancer-associated fibroblasts [21, 22]. Alarmingly, the use of anxiolytic Lorazepam, which has off-target agonism of GPR68, but not alprazolam, which does not activate GPR68, has recently been associated with a 3.8-fold higher rate of pancreatic cancer progression and related death than in non-users [23]. Additionally, lorazepam use was correlated with significantly worse overall survival in prostate, ovarian, head and neck, uterine, colon, and breast cancer, and melanoma [23, 24]. Here, we describe the discovery of a novel class of small molecule GPR68/OGR-1 inhibitors named ogremorphins, and using this class, we show that GPR68-ATF4 signaling is a novel glioblastoma pro-survival pathway activated in an autocrine manner by extracellular protons. Moreover, we show that genetic and pharmacological disruption of GPR68 signaling in glioblastoma cells induces ferroptosis, an iron-mediated cell death program, across a diverse set of GBM lines.

## Materials and methods

### Chemical screen

All zebrafish experiments were approved by the Vanderbilt University Institutional Animal Care and Use Committee. The chemical screen for small molecules that perturb the embryonic development in zebrafish was performed as described previously [25, 26]. Briefly, pairs of wild-type (WT) zebrafish were mated, and fertilized eggs were arrayed in 96-well microtiter plates (5 embryos/well) containing 100 µL E3 water. At 4 h post fertilization (hpf), a small-molecule library from the High Throughput Screening Facility was added to each well to a final concentration of 10 µM. Embryos were incubated at 28.5 °C and examined for gross morphological changes indicative of reproducible embryonic defects at 24 and 48 hpf. A total of 30,000 compounds were screened.

### Alcian blue staining

Staged embryos and larvae were anesthetized with tricaine and sacrificed by immersion in 4% formaldehyde (prepared from paraformaldehyde and buffered to pH 7 in phosphate-buffered saline). Fixed animals were rinsed in acid–alcohol (0.37% hydrochloric acid, 70% ethanol), and stained overnight in Alcian blue. After destaining in several changes of acid–alcohol, preparations were rehydrated. Following rinsing and clearing in a solution of 50% glycerol and 0.25% potassium hydroxide, cartilage was visualized under a stereomicroscope.

### Generation of U87 pHluorin2-GPI cell line

U87 cells were transfected using Lipofectamine 3000 with pTol2 (Ubi: pHluorin2-GPI) (VectorBuilder; vector ID vb200601-1084rcb), and pCMV-Tol2 (Addgene:31,823). Three weeks post-transfection, cells were flow sorted for pHluorin2-GPI expression and clonally selected.

### Alkalization assay

pHluorin2-GPI cells in HEPES-buffered FluoroBrite medium (ThermoFisher) were imaged using 488 nm excitation/525 nm emission using the Lionheart Imager (BioTek). Alkaline medium was added to the well using the automated injection system to adjust the pH of the well to pH 8.4 and imaged with the same settings after 20 s.

### In vitro cell viability assays

GBM neurospheres and low passage PDX models were plated in 96-well plates at 10,000 cells per well in 50 µL of neural stem cell media. The next day, the cells were treated with OGM compounds at the indicated concentrations, in triplicates, by adding an equal volume of medium containing 2x the final concentration of the compound. Following 72 h of incubation under standard cell culture conditions, relative cell number was assessed using Cell Titer-Glo Luminescent Cell Viability Assay (Promega, Madison, WI, USA) following the manufacturer’s instructions. Luminescence was determined using a Cytation 5 reader and Gen5 software package (BioTek, Winooski, VT, USA). For U87 and U138 cell lines, one thousand cells were plated per well in a standard 96-well plate and allowed to attach for 24 h before exposure to concentrations of vehicle, OGM, or TMZ. Cells were treated for 72 h and then stained with DAPI. A 10× magnification lens on a LionheartFX (BioTek) was used to image the wells, and images were stitched together with Gen5 software (BioTek). Automated nuclei counting was also done using the Gen5 software. Results reported as a percent response relative to DMSO control. IC50 was determined by GraphPad Prism version 6.07.

### GBM spheroid assay

One thousand cells per well were plated in an ultra-low attachment, round-bottomed, 96-well plate, and spheroids were allowed to form for 3 days. Wells were then exposed to concentrations of vehicle, OGM or TMZ or a combination for 3 days. Spheroids were imaged in bright-field at 10× using z-stacks that were collapsed into z-projections in the Gen5 software using the LionheartFX (BioTek). Automated measurements of the spheroid area were obtained using Gen5 software.

### Western blot analysis

Using Pierce ^TM^ BCA Protein Assay Kit (CAT # 23,227, ThermoFisher Scientific, Waltham, MA, USA), protein concentrations were determined for each sample following the manufacturer’s protocol. Gel electrophoresis was conducted on NuPAGE ^TM^ 4–12% Bis-Tris gels (CAT # NP0321BOX, Invitrogen, Waltham, MA, USA) using 20 µg of total protein per sample. Proteins were transferred from Gel to PVDF membrane by semi-dry transfer. Membranes were blocked using Intercept® (PBS) Blocking Buffer (CAT #927-70001, LI-COR, Lincoln, NE) for one hour at room temperature on a tilting shaker. Primary antibodies in 5% non-fat dry milk were added to the membranes for overnight incubation at 4 °C on a rotating shaker. Primary antibodies included Transferrin receptor (TFRC) (CAT # 13-6800), heme-oxygenase 1 (HO-1) (CAT # 5853 S), activating transcription factor 4 (ATF4) (D4B8; CAT # 11,815), ChaC glutathione specific gamma-glutamylcyclotransferase 1 (CHAC1) (OTI1E2), caspase 3 (CAT # 9662), and cleaved caspase 3 (CAT #9661S) with GAPDH (1D4) and α-tubulin (CAT # 32-2500) used as normalization controls. The next day, membranes were washed in three consecutive five-minute intervals with PBST (Tween 0.5%). Corresponding secondary HRP-conjugated antibodies (CAT # NB300-221, CAT # G21234, CAT # A16078) in 5% non-fat dry milk were added to incubate at room temperature for one hour on a tilting shaker. The membranes were washed with PBST in four, five-minute intervals before protein visualization using Radiance Q (CAT # AC2101, Azure Biosystems, Dublin, CA, USA) on Bio-Rad ChemiDoc ^TM^ MP Imaging System (Hercules, CA, USA). For multiple proteins, blots were cut and/or stripped with Restore Western Blot Stripping Buffer (CAT # 21,063, Thermo Scientific) for 30 min and re-blotted as described. Protein quantification was completed in triplicate using Fiji.

### PDX culture

The neurospheres were provided to us by Drs. Angelo Vescovi, Jeremy Rich, and Ichiro Nakano. The PDX models were acquired from Dr. Jann Sarkaria, from the PDX National Resource at the Mayo Clinic. All neurosphere lines and PDX models have were tested for mycoplasma contamination and identified by STR analysis before the beginning of the study. For experimentation, PDX and neurosphere lines were cultured in neural stem cell medium consisting of KnockOut DMEM/F-12 supplemented with StemPro NSC SFM Supplement, bFGF, EGF, L-glutamine, and Penicillin/Streptomycin.

### GPR68 knockdown with siRNA

siRNAs for knockdown were obtained from Dharmacon. GPR68-siRNA1 (CAT # D-005591-01-10) and GPR68-siRNA2 (CAT # D-005591-02-0010). For controls, we used siGENOME non-targeting siRNA Control Pool standard non targeting siRNA was used (Dharmacon, CAT # D-001206-13-10). Cells were reverse transfected with 10 ng of siRNA using lipofectamine RNAiMAX (Thermo Fisher, CAT # 13,778,150), in a 12-well plate (CELLTREAT Scientific Products, 229,111).

### Knockdown of GPR68 CRISPRi

Cells were reverse transfected on a 12 well cell culture dish with 2.5 µg dCas9 per well. The next day, fresh media was added to the wells, and the cells were transfected with 12 pmol sgRNA using lipofectamine RNAiMAX. Alt-R modified sgRNAs were obtained from IDT targeting sequences were GPR68 sgRNA1: 5’-ACCGCCAUCCUGUUUAUAGA-3’, and GPR68 sgRNA2: 5’- GAAGGGGCCACACUCCUCAU-3’, GPR68 sgRNA3: 5’-CCAUACCAUCCACCAGACGC-3’, and GPR68 sgRNA4: 5’-GCCCCUUCAGGCCCAAAGAU-3’.

### Overexpression of ATF4

Cells were reverse transfected in a 12 well cell culture dish with 2.5 µg plasmid per well or 20 µg per 100 mm cell culture dish using lipofectamine 3000. Plasmids were acquired from VectorBuilder Inc. Human ATF4 was overexpressed using VB230104-1203pag. Alternatively, VB900139-8319ega, a control plasmid with a fragment of E. coli beta-galactosidase, was used as a transfection control. Cells were grown and collected for westerns, Liperfluo, or CellTiter-Glo experiments.

### RNA-seq

913 and 08-387 cells (1 million each) were treated with 0.5 µM OGM, and Mayo6 and Mayo39 cells were treated with 2 µM for 72 h. Cells were trypsinized and flash frozen. Cell pellets were sent to Azenta (Chelmsford, MA) for RNA-isolation. All RNA samples had RIN between 7.7 and 10.0, and sequencing, 20–30 Million reads on Illumina HiSeq, PE 2 × 150 bp. Read counts were normalized and differential expression was determined using DESeq2. Gene Set Enrichment Analysis was done on DAVID.

### Glutathione assay

GSH-Glo™ Glutathione Assay (Promega) was performed according to manufacturer protocol. Briefly, U87 cells were plated at 10,000 cells per well in a 96 well plate. The following day, cells were treated with DMSO as a control vehicle or 2 µM OGM for 24 h. Medium was removed from wells and 100 µL GSH-Glo™ Reagent was added to each well and incubated on a plate shaker for 30 min. 100 µL Luciferin Detection Reagent was added to each well and mixed briefly. After 15 min of incubation, luminescence was detected on the Promega GloMax luminometer.

### In vitro staining for immunofluorescence

Mitotracker, Lysotracker, TMRM and Hoescht dyes were obtained from Invitrogen™ and used according to manufacturer’s protocols.

### Liperfluo

Cells were plated on a 100 mm cell culture dish and incubated overnight at 37^o^ C in 5% CO_2_. Media was then replaced with 30 mL of fresh media with DMSO, OGM, or Erastin (APExBIO Technology, LLC, CAT # B1524) and cells incubated for 3 days. On the third day, 3 mL fresh media with 2.5 µM Liperfluo (Dojindo Molecular Technologies, Inc, CAT # L248-10) resuspended in DMSO was added and cells were incubated at 37^o^ C in 5% CO_2_ for 1 h. Cells were subsequently trypsinized for 5 min, pelleted by centrifugation, and resuspended in cell sorting media (1% BSA and 1 mM EDTA in PBS pH 7.4). Ten thousand events were recorded on a BD LSR II and the data processed using the FlowJo software.

### ATF4 and CHAC1 knockdown

Cells were reverse transfected on a 12 well cell culture dish with 2.5 µg dCas9 per well. The next day, fresh media with or without 2 µM OGM was added to the wells, and the cells were transfected with 12.5 pmol sgRNA using lipofectamine RNAiMAX (Thermo Fisher). Three days later, cell survival was assessed by lysing the cells with 1x Passive lysis buffer and quantification with CellTiter-Glo. Alternatively, total RNA was collected after three days of treatment, for cDNA generation and qRT-PCR. Alt-R modified sgRNAs were obtained from IDT targeting sequences ATF4 sgRNA1: 5’-GAUGUCCCCCUUCGACCAGU-3’, ATF4 sgRNA2: 5’-GCGGUGCUUUGCUGGAAUCG-3’, ATF4 sgRNA3: 5’-CCACCAACACCUCGCUGCUC-3’, ATF4 sgRNA4: 5’-AGCUCAUUUCGGUCAUGUUG-3’, ATF4 sgRNA5: 5’-AAUGAGCUUCCUGAGCAGCG-3’, *CHAC1* sgRNA1: 5’- ACGGCGACCCUCAAGCGCUG-3’, *CHAC1* sgRNA2: 5’- GAACUGAGCGGACGGCGUAG-3’, *CHAC1* sgRNA3: 5’-UGUGCCAGGCACCAUGAAGC-3’, *CHAC1* sgRNA4: 5’- UGCUUCAUGGUGCCUGGCAC-3’, and *CHAC1* sgRNA5: 5’- GACUCCUGCUUCAUGGUGCC-3’.

### Transmission electron microscopy (TEM)

U87 cells were treated with either DMSO or 2 µM of OGM for 12 or 24 h. Cells were fixed with 2.5% glutaraldehyde. Electron Microscopy Core Imaging Facility at UMB prepared samples for TEM imaging after fixation. Samples were imaged on FEI Tecnai T12.

### Compounds

OGM was resynthesized as described in Fig. S2 (Jubilant Biosystems, India) and structure validated by NMR and LCMS in Fig. S3. Temozolomide was purchased from TOCRIS bioscience (Cat No. 2706). NE 52-QQ57 was purchased from Selleckchem (Houston, Texas).

### Statistics

Were appropriate ANOVA, or student’s T-test were conducted in PRISM. Chi squared T(X) was used for the Liperfluo analysis. A value T(X) > 4 implies that the two distributions are different with a *p* < 0.01 (99% confidence). For drug interaction and therapeutic interaction, the coefficient of drug interaction (CDI) is calculated as follows: **CDI = AB/(A×B)**. According to the impact of each group, AB is the value of combined treatment and A or B values are the value of the single agent group. Thus, CDI < 1, = 1 or > 1 indicates that the drugs are synergistic, additive, or antagonistic, respectively. CDI < 0.7 indicates that the drug is significantly synergistic.

## Results

### Identification of a highly specific GPR68 inhibitor

**Fig. 1:**
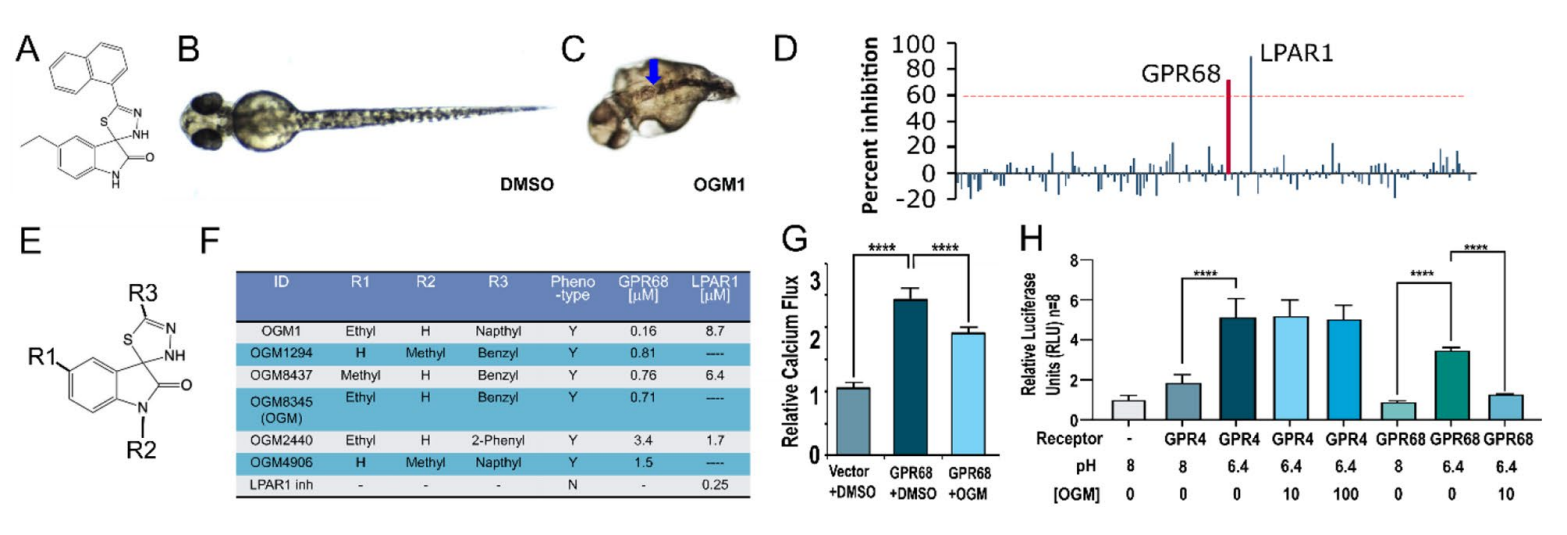
Ogremorphin is a highly specific inhibitor of GPR68. (**A**) Structure of OGM1 (5-ethyl-5’-(1-naphthyl)-3’H-spiro [indole-3,2’- [1, 3, 4]thiadiazole]-2-one). (**B** and **C**) Dorsal view of vehicle (DMSO)- and 10 µM OGM1-treated zebrafish embryo at 48 h post-fertilization (hpf). In contrast to the control embryo, OGM1-treated embryo showed abnormal melanocyte pigmentation, characterized by a striped pattern (blue arrow) restricted to the dorsal aspects of the embryo. (**D**) OGM1 only inhibited 2 GPCRs in a screen of 158 GPCRs (Data in Supplemental Table 2). (**E** and **F**) Core scaffold for OGM derivatives and structure activity relationship (SAR) analysis. Loss of LPAR1 activity did not correlate with loss of the zebrafish phenotype. Commercial inhibitor (inh) of LPAR1 (Ki16425, Sigma) also failed to recapitulate the phenotype. (**G**) Acidic stimulation of GPR68 expressed in HEK293 elicits a calcium response that is inhibited by OGM (N = 8). (**H**) Serum-responsive element-luciferase (SRE-luc) reporter by itself had low basal activity in 293T cells. Upon co-transfection with GPR4, luciferase activity increased with acidification but was not inhibited by OGM at 1, 10, or 100 µM. By contrast, when GPR68 was co-transfected with SRE-luc and stimulated by acidification, 10 µM OGM completely inhibited the signal. (n = 4, ****P < 0.0001)

To discover new potential regulators of cancer, we turned to developmental processes, as key regulators of embryonic development are known to play critical roles in cancer [27–30]. We conducted an unbiased chemical genetic screen of ~ 30,000 compounds to identify novel small molecules that selectively perturb zebrafish development [25, 26, 31, 32]. This screening effort led to the identification of 5-ethyl-5’-(1-naphthyl)-3’H-spiro [indole-3,2’- [1, 3, 4]thiadiazole]-2-one, herein named ogremorphin-1 (OGM1) (Fig. 1A). OGM1 induced phenotypes encompassing a wavy notochord, abnormal pigmentation, craniofacial defects, ventral curvature, and a shortened body axis (Fig. 1B, C, S1A). While the disruptions in melanophore and craniofacial cartilage development are consistent with defects in neural crest development, the wavy notochord is consistent with dysregulation of copper metabolism [33].

To identify OGM1’s candidate target, we profiled it for binding across a panel of 442 kinases (KinomeScan, DiscoveRx) and assessed its activity against 158 GPCRs in a single-point assay in Chem-1 cells that uses a promiscuous G_α15_ protein to trigger calcium flux (Table S1, 2) (GPCR Profiler, Millipore) [34, 35]. In our profiling studies, OGM1 did not physically interact with the kinase domain of any kinase. Remarkably, OGM1 displayed significant inhibitory activity exclusively against two targets: the lysophosphatidic acid receptor 1 (LPAR1), and extracellular proton-sensing GPR68/OGR1 (Fig. 1D; Table S2) [17].

Since the zebrafish phenotype caused by OGM1 was not consistent with published LPAR1 knock down in zebrafish, [36] we assessed if loss of GPR68 activity was sufficient to cause the phenotypes seen in OGM1-treated zebrafish. We used morpholino oligonucleotides to knock down GPR68 expression and observed dose-dependent neural crest–specific phenotypes in melanocytes and craniofacial cartilage using 1.5 ng and 3 ng morpholino, whereas the same amount of the mismatched morpholino did not recapitulate these phenotypes (Fig. S1A, B, S2). We also observed the same phenotype in F0 embryos in which the *GPR68* gene was targeted by CRISPR/Cas9 (Figs.  S1A, B, S2). Finally, GPR68 knockdown/knockout recapitulated the OGM1-induced wavy notochord and short body-axis phenotypes (Fig. 1D, H). These results suggest that the OGM1-induced phenotypes are specifically due to inhibition of GPR68.

To validate which GPCR was involved in this phenotype, a chemical genetic segregation analysis was carried out in zebrafish embryos [31]. A small-scale structure-activity relationship study around the core spiro[1H-indole-3,2’-3 H-1,3,4-thiadiazole]-2-one pharmacophore generated 3 molecules that were similar to OGM1 but lacked LPAR1 activity (Fig. 1E, F) [37]. The GPR68 inhibitory activity of the analogs segregated with the ability to induce the zebrafish phenotype. Furthermore, commercially available inhibitors of LPAR1 (Ki16425) failed to induce the phenotype at concentrations up to 50 µM, ~ 200X its IC_50_. This validates the results of our genetic findings, that GPR68, causes the phenotype observed with OGM treatment. Given its sub-micromolar potency and GPR68 selectivity, one of the OGM1 analogs, OGM8345 (henceforth called OGM), was resynthesized and used for further experiments (Fig. S3, S4).

To assess the specificity of the calcium response with extracellular acidification, we transfected GPR68 into human embryonic kidney (HEK293) cells, which normally do not express GPR68. The GPR68-transfected cells had a significantly greater calcium response than vector transfected control; OGM significantly reduced this response (Fig. 1G). Besides GPR68, the other members of the proton-sensing GPCR family are GPR4 and GPR65 [17, 38]. Notably, the interspecies homology of orthologs hs.GPR68 and dr.GPR68, is significantly greater than that of human paralogs hs.GPR4 and hs.GPR65 (Fig. S5A, B). Because GPR4, the paralog with highest homology with GPR68, was not covered in our initial GPCR profiling, we tested whether OGM could inhibit GPR4 by examining the effects of OGM on acid-induced serum responsive element (SRE) luciferase activity in HEK293 cells transfected with either GPR4 or GPR68. As previously reported, mild acidification increased luciferase activity in GPR4 and GPR68 transfected cells above that of vector control (Fig. 1H) [39]. Signaling was inhibited only in cells transfected with GPR68 (Fig. 1H), demonstrating that OGM is a selective and specific inhibitor of GPR68. These data show OGM is a highly specific inhibitor of GPR68, which is a pH sensing receptor that is activated by the range of pH seen in the tumor microenvironment and hypothesized to be a potential therapeutic target for other cancers [20].

### Glioblastoma senses acidification through GPR68

**Fig. 2:**
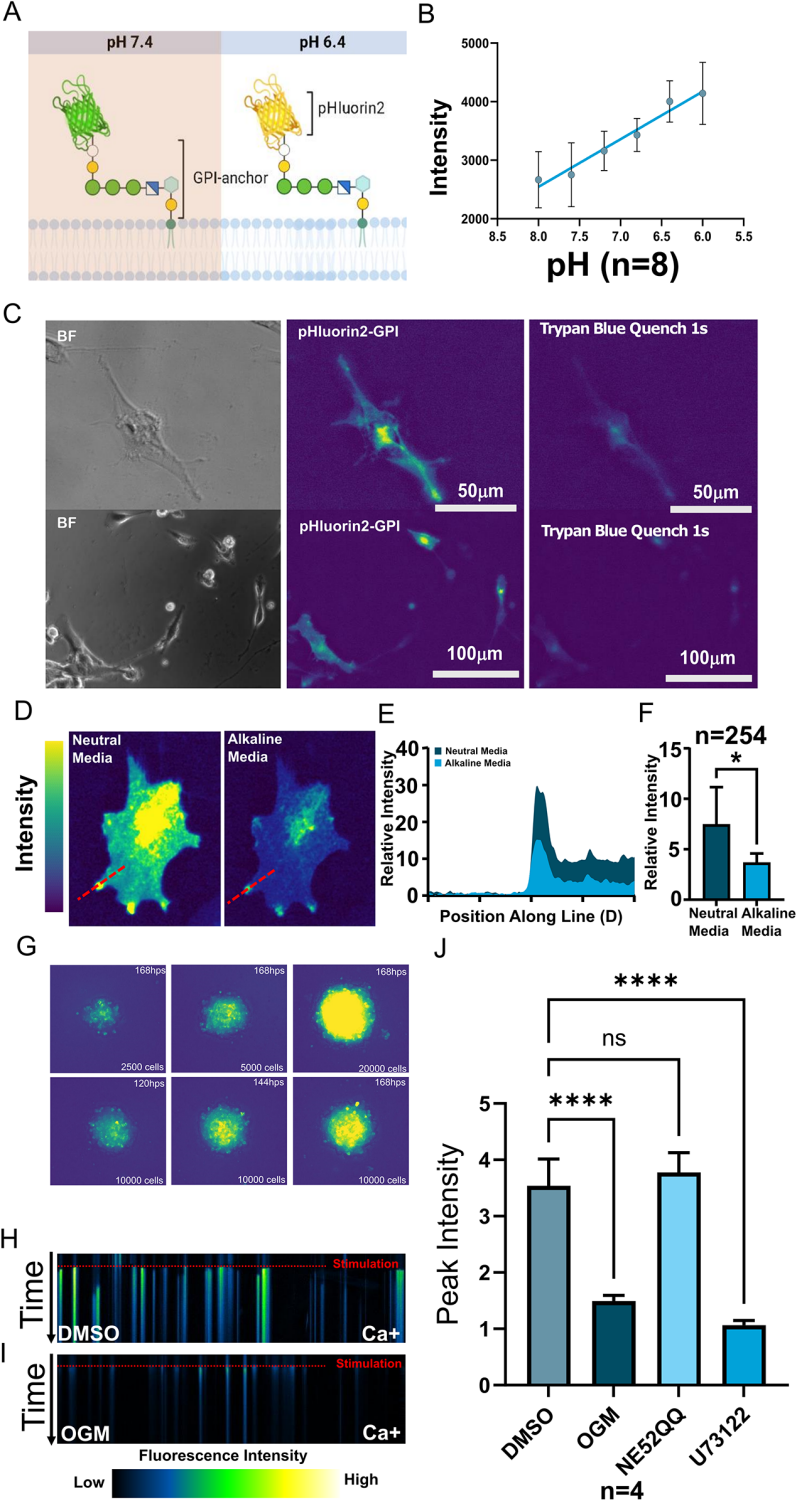
Glioblastoma activates GPR68 by acidifying their extracellular milieu. (**A**) Schematic representation of the extracellular pH reporter GPI-anchored pHluorin2 (pHluorin2-GPI), which increases in fluorescence intensity upon acidification. (**B**) Strong correlation between fluorescence intensity and extracellular pH in cells stably expressing pHluorin2-GPI, imaged at 469 nm excitation/525 nm emission. (**C**) pHluorin2-GPI fluorescence was quenched by the vital dye trypan blue, which is excluded from live cells, confirming that the visualized acidic micro-domains are extracellular. (**D**) U87 glioma cells expressing pHluorin2-GPI reporter exhibited higher-intensity fluorescence, particularly at cellular protrusions in neutral pH media. Fluorescence was markedly attenuated within 20 s of buffering to pH 8.4 (After), confirming the correlation of fluorescence intensity with low extracellular pH. (**E**) Quantification of fluorescence intensity along the red line in (**A**) confirmed a drastic reduction at pH 8.4. (**F**) The overall cellular intensity of the pHluorin2-GPI signal was reduced by the addition of an alkaline buffer (*P* < 0.05, n = 6). (**G**) When grown in spheroids, the extracellular acidification increases over time and becomes more organized (**H, I**) Kymograph of U87 cells (**H**) responding to acidification (stimulation) with calcium release. OGM (**I**) greatly attenuated acid-induced calcium release, in contrast to DMSO vehicle control. (**J**) Peak calcium responses of U87 cells to acid stimulation in the presence of the GPR68 inhibitor OGM, the GPR4 inhibitor NE52-QQ or the PLC inhibitor U73122 reveals that the response is mediated specifically by the GPR68-PLC pathway, but not by GPR4 (N = 6 OGM, *P* < 0.0001; NE52QQ, not significant; U73122, *P* < 0.0001)

As in many solid cancers, the low extracellular pH of the TME of GBM promotes glioblastoma survival and chemoresistance [19, 40]. To visualize changes in the GBM acidic milieu we generated a Glycosylphosphatidylinositol (GPI) anchored pHluorin2 (Fig. 2A). The GPI anchor is a posttranslational modification to a protein that attaches it to the outer leaflet of the cell membrane, and pHluorin2 is a fluorescent protein which upon acidification, emits increased fluorescence following 469 nm excitation (Fig. 2A, B) [41, 42]. We used U87 glioblastoma cells, which highly express *GPR68*, to generate clones that stably expressed pHluorin2-GPI under a ubiquitous promoter. In these cells, the pHluorin2 fluorescence was quenched by Trypan blue (Fig. 2C). Since Trypan Blue is a vital stain which is excluded from entry into live cells, this result indicates that the acid-responsive fluorescence originates from the extracellular space. The pHluorin2-GPI-expressing U87 cells displayed foci of high-intensity fluorescence particularly in lamellipodia and filopodia, which were significantly attenuated in alkaline buffered media (pH 8.4) (Fig. 2D-F). These results support the existence of extracellular zones of local acidification on the surface of cells cultured in globally neutral pH conditions.

The in vitro 3D spheroid model, in which cancer cells are grown as aggregates, is thought to recapitulate many aspects of the TME, including the nutrient, oxygen and pH gradients that exist in solid tumors in vivo [43]. When 3D spheroids were generated from the U87 cells, extracellular acidification, as indicated by the pHluorin2-GPI fluorescence, was observed throughout the spheroids (Fig. 2G, S6A, B). After 72 h of spheroid formation, acidic domains appear to stabilize within the central core of growing tumor spheroids. This is consistent with previous findings that even well-oxygenated regions of tumors are acidic and that acidic regions of tumors extend beyond their hypoxic core [44, 45]. These results mirror the clinical characterization of highly acidic GBM tumor cores, and the change in the pH dependent fluorescence of the reporter, pHlourin2, demonstrates acidification of the extracellular milieu by GBM. To determine if GBM cells respond to their own acidic extracellular milieu as a form of autocrine signaling, we measured calcium release in response to acidification. GPR68 is known to couple to the G_q_ subunit, which acts to release calcium from the ER in a PLC dependent pathway. We shifted the medium from pH 7.8 to 6.4 triggering a rapid and robust calcium flux, as measured by the fluorescence intensity of calcium-sensitive dye Cal520-AM (Fig. 2H). This calcium flux was blocked by OGM and PLC inhibitor U73122 (Fig. 2H, I, J) [46, 47]. This data suggests GPR68 is activated in GBM cells in an autocrine manner, in response to the extracellular acidification generated by the GBM cells themselves.

### Loss of GPR68 activity reduces GBM survival

**Fig. 3:**
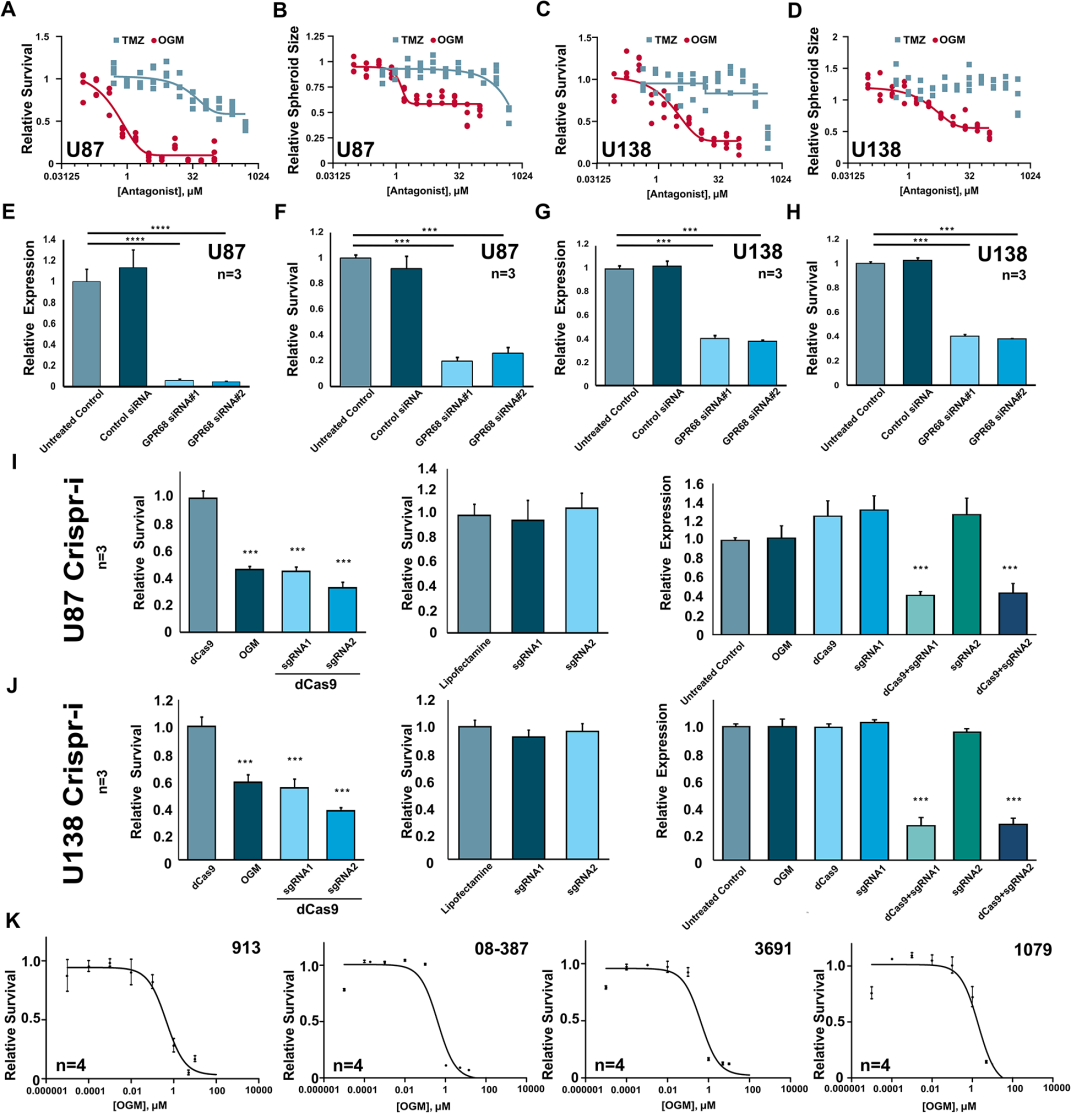
GPR68 regulates cell survival in glioblastoma. (**A**) OGM is a more potent inhibitor of U87 cell growth in 2D cell assay than Temozolomide (TMZ). (**B**) OGM is a more potent inhibitor of U87 spheroid growth than TMZ. (**C**) OGM, but not temozolomide (TMZ), caused dose-dependent inhibition of U138 cell growth in 2D culture. (**D**) OGM, but not TMZ, significantly decreased the growth of U138 3D spheroids. (**E, F**) siRNAs targeting GPR68 in U87 cells reduced GPR68 expression and reduced cell survival, whereas control siRNA had no effect on either. (**G, H**) siRNAs targeting GPR68 in U138 cells reduced GPR68 expression and reduced cell survival while control siRNA had no effect. (**I**) CRISPRi targeting GPR68 in U87 cells reduced both survival and expression of GPR68, while sgRNA alone and dCas9 alone have no effect on survival or expression. (**J**) CRISPRi targeting GPR68 in U138 cells reduced both survival and expression of GPR68, while sgRNA alone and dCas9 alone have no effect on survival or expression. (**K**) OGM reduced survival of 4 different PDX lines in 2D cell survival assays

Although others have primarily characterized the role of GPR68 in cancer associated fibroblasts, we hypothesize that in GBM, GPR68 mediates pro-survival mechanisms triggered by the acidic TME. Consistent with this, OGM treatment decreased viability of U87 cell more potently than temozolomide (TMZ), the first-line chemotherapy for GBM (Fig. 3A). In the 3D spheroid model of U87, which we observed to create a greater acidic core, OGM continued to exhibit even greater potency than TMZ which was less effective in the spheroid model (Fig. 3B). Furthermore, OGM treatment decreased viability of U138 glioblastoma cells, which are resistant to TMZ, both in monolayer (Fig. 3C) and 3D spheroid models (Fig. 3D). Interestingly, OGM and TMZ demonstrated strong synergistic killing of PDX 08-387 cells with a coefficient of drug interaction (CDI) < 0.7 (CDI < 1 supports synergism; Fig. S7A-C). Taken together this data suggests OGM is more potent than TMZ at killing GBM cells, but combinatorial therapy with TMZ may be more improve TMZ efficacy.

To confirm that the effect of OGM on glioma cells is due to GPR68 inhibition, we knocked down GPR68 using siRNA in U87 and U138 cells. Two GPR68-targeting siRNAs, which significantly decreased GPR68 transcript levels (Fig. 3E), also significantly decreased U87 viability, compared to the control siRNA (Fig. 3F). Additionally, the GPR68-targeting siRNAs significantly decreased U138 viability, compared to the control siRNA (Fig. 3G, H). Furthermore, knockdown of GPR68 using CRISPR interference (CRISPRi), reduced GPR68 expression and decreased cell viability in U87 and U138 cells, while neither the dCas9 alone nor the respective sgRNAs alone had any effect (Fig. 3I, J; Fig. S8A-D). Therefore, the reduction in GBM viability by OGM mediated inhibition of GPR68 activity is recapitulated by siRNA and CRISPRi-mediated knockdown of GPR68 expression.

Because glioblastoma cell lines like U87 and U138 can lose some characteristics of primary GBM tumors while in long-term culture, patient-derived xenograft (PDX) cell models are considered superior models that faithfully maintain the genomic and pathologic features found in the primary tumors [48]. In 2D monolayer cultures, OGM treatment significantly reduced viability of each of the 6 independent patient derived lines (PDX and Neurospheres), with LC_50_’s in the 0.42 to 2.7 µM (Fig. 3K, S8E). In 3D spheroid models, OGM treatment significantly reduced viability of the PDX lines with a similar LC_50_ range (Fig. S8F). Similarly, OGM treatment significantly reduced the viability of mouse glioblastoma line GL261 (Fig. S8E). Overall, OGM treatment potently reduced viability of all 13 GBM cell lines tested (Fig. S9). By contrast, OGM had no effect on HEK293 cell viability, and did not induce excess cell death in zebrafish larvae (Fig. S10A-D), ruling out nonselective toxicity of OGM. In Tg(neuroD1: EGFP) zebrafish larvae treated with OGM, no significant increase in cell death was observed in non-neural (GFP-) cells or in neural (GFP+) cells, which include neurons and glial cells (Fig. S10E-F). These results suggest that OGM acts specifically on Glioblastoma cells with no overt toxic effects on normal or neuronal tissues, across species and subtypes.

Additionally, spheroids grown in acidic media (pH 6.2) were larger and grew faster than those grown in basic media (pH 8.0) (Fig. S11A, B). Similarly, spheroids treated with Ogerin, a positive allosteric modulator of GPR68, grew faster than controls (Fig. S11C) [49]. Furthermore, cells cultured in acid were more susceptible to OGM inhibition (Fig. S11D, E). These results suggest that, in response to acidic extracellular milieu, GPR68 mediates both pro-survival and pro-growth pathways conserved in GBM cells, and that OGM selectively inhibits this pathway to kill GBM.

### OGM triggers ferroptosis in GBM cells

**Fig. 4:**
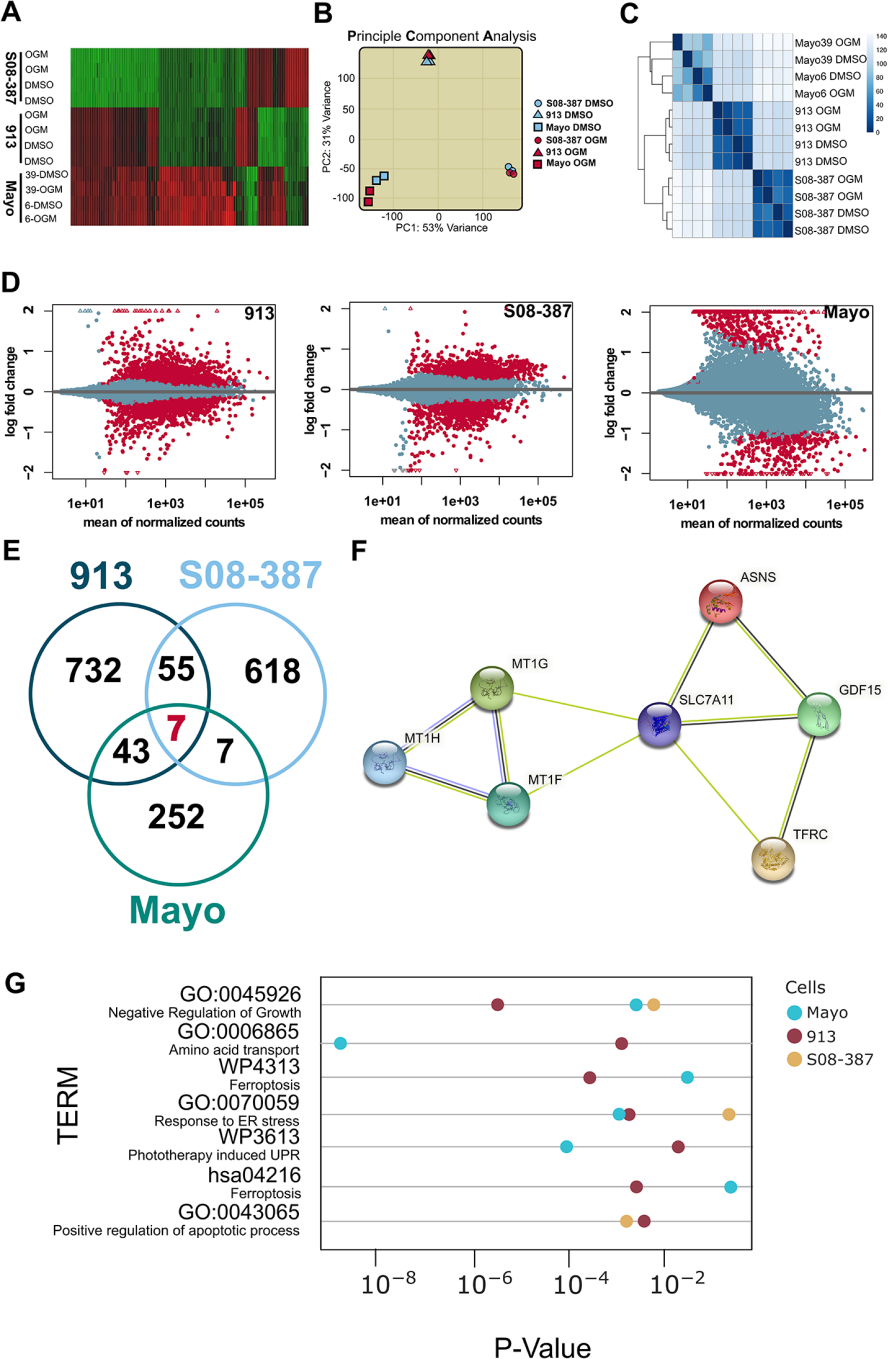
RNA-seq of PDX GBM indicates OGM induces ferroptosis. (**A**) Heatmap of gene expression changes shows broad changes in transcriptomes in PDX cells after treatment. (**B**) PCA comparison of transcriptomes demonstrates that differences across cell types are greater than differences induced by OGM treatment. This difference is also shown through hierarchical clustering in (**C). (D**) SDE genes from 913, 08-387, and Mayo PDX cells after OGM treatment were 837, 687, and 309, respectively (FDR < 0.01). (**E**) Comparison of SDE genes found that only 7 common genes were dysregulated in all three treatment groups. (**F**) String analysis of 7 commonly dysregulated genes. (**G**) Subset of Gene set enrichment analysis of terms implicates ferroptosis as a mechanism of cell death (full analysis in Fig. S12)

To understand the molecular mechanisms by which OGM causes GBM cell death, we performed a global transcriptomic profiling (RNA-seq) of four independent, molecularly heterogeneous, human GBM patient derived cell lines 913, 08-387, Mayo6 and Mayo39 treated with DMSO vehicle or OGM at respective LC_50_’s for 72-hours. The differential gene expression analysis revealed significant transcriptomic changes with OGM in all lines (Fig. 4A; Table S3). The principal component analysis (PCA) indicated that each GBM cell line was significantly different from each other (Fig. 4B), consistent with known molecular heterogeneity of GBM cells [5]. Moreover, each OGM-treated cell line was transcriptionally most similar to its untreated counterpart, suggesting that the underlying differences between glioblastoma cell lines are greater than the changes induced by the OGM-treatment (Fig. 4B, C). Next, we identified significantly differentially expressed (SDE) genes (ABS Log FC 0.585, False discovery rate (FDR) < 0.01) induced by the OGM-treatment for each GBM cell line (Fig. 4D; Tables S4-6). A Venn diagram highlights the 7 SDE genes that were consistently differentially expressed in OGM treatment across the different GBM types (Fig. 4E, F; Table S7).

Given the substantial differences in the baseline transcriptomic landscape across different GBM types, and the relatively small differences between treatment and control groups for each line, we sought to identify dysregulated pathways that were shared. When SDE genes in each group were subjected to GO, KEGG and WIKIPATHWAY gene set enrichment analysis (Fig. S12; Table S8), it revealed “Negative Regulation of Growth” (GO:0045926), “Amino Acid Transport” (GO:0006865), “Ferroptosis” (WP4313, has:4216), “Unfolded Protein Response (UPR)/Endoplasmic Reticulum (ER) Stress” (GO:0070059, WP3613), and “Positive Regulation of Apoptotic Process” (GO:0043065) as shared enriched terms (Fig. 4G). Notably, 3 of the 7 SDE genes induced by the OGM-treatment, *ASNS, GDF15*, and *SLC7A11*, are each annotated as a marker of ferroptosis in the FerrDB database [50–52].

**Fig. 5:**
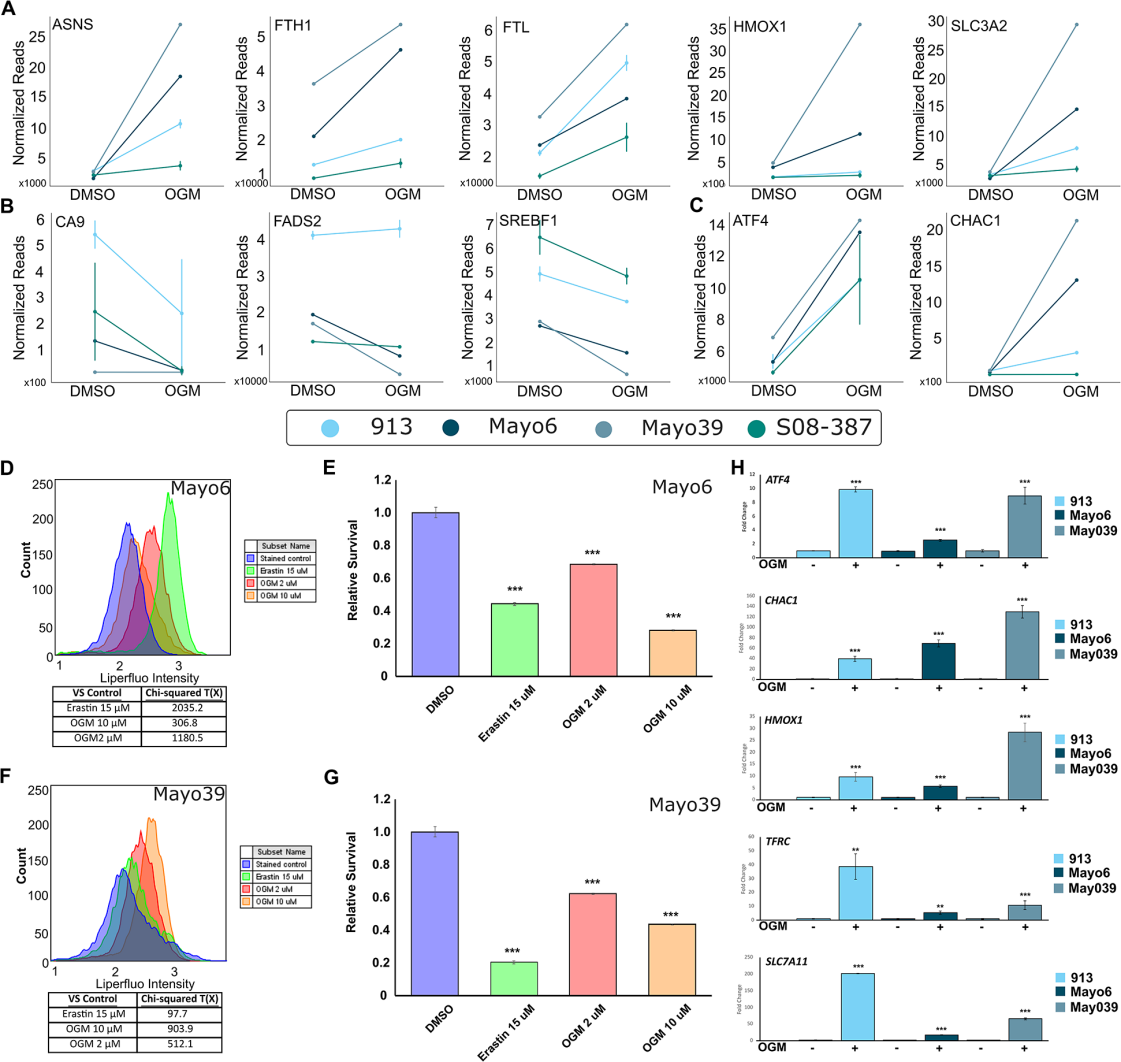
OGM causes ferroptosis in PDX models of GBM. (**A**) Known markers and mediators of ferroptosis were increased in PDX cells treated with OGM. (**B**) Known suppressors of ferroptosis were decreased in PDX cells treated with OGM. (**C**) ATF4 and CHAC1, involved in both ferroptosis and ER stress response, were increased in OGM treatment groups. (**D**) OGM and Erastin demonstrated very strong for induction of lipid peroxidation in Mayo6 cells. All treatments were highly significant with Chi-squared > 4, which is equal to *p* < 0.01) (**E**) At the concentrations used OGM and Erastin both caused significant cell death (**F**) OGM and Erastin demonstrated very strong induction of lipid peroxidation in Mayo39 cells. All treatments were highly significant with Chi-squared > 4, which is equal to *p* < 0.01) (**G**) At the concentrations used OGM and Erastin both caused significant cell death (**H**) OGM induced key markers of ferroptosis ATF4, CHAC1, HMOX1, TFRC, and SLC7A11, confirmed via qPCR in PDX lines

Ferroptosis is an iron-dependent programmed cell death pathway characterized by an accumulation of lipid peroxides that is genetically and biochemically distinct from other programmed cell death mechanisms [53–55]. Consistent with the induction of ferroptotic cell death in GBM cells, OGM treatment significantly altered expression of 3 of the genes encoding metallothioneins (MTs), which directly bind iron to protect cells from oxidative damage (Fig. 4F) [56, 57]. A closer examination of the RNA-seq data revealed a trend that OGM-treatment induced expression of several classic ferroptosis markers, specifically *TFRC, ASNS, FTH1, FTL, HMOX1* and *SLC3A2* (Fig. 5A) [50, 54, 55, 58–63]. Moreover, there was also a trend showing that OGM treatment significantly reduced expression of known ferroptosis suppressors *CA9*, *FADS2*, and *SREBF1* (Fig. 5B) [64–66]. Additionally, OGM-treatment induced expression of *ATF4* and *CHAC1*, the core mediators of both ferroptosis and ER stress (Fig. 5C). This data is consistent with ferroptosis induces such as Erastin which have been shown to up-regulate ATF4 in multiple cell types including U87 [52, 67–70]. Although commonly associated with ER stress, ATF4 can also induce ferroptosis as a key transcription factor that induces expression of *CHAC1* [71, 72]. *CHAC1* encodes ChaC (also Glutathione Specific Gamma-Glutamylcyclotransferase-1), which degrades glutathione (GSH), the main antioxidant mechanism in cells, resulting in accumulation of toxic lipids [54, 71–73]. Consistent with the RNA-seq analysis of OGM-treated PDX models that suggested ferroptosis induction, PDX cells treated with OGM had increased levels of lipid peroxidation and were also sensitive to ferroptosis inducer Erastin (Fig. 5D-G). Moreover, OGM-induction of ferroptosis markers ATF4, CHAC1, HMOX1, and TFRC were confirmed by qPCR in PDX cells (Fig. 5H). These results indicate that OGM treatment causes GBM cell death via ferroptosis.

To confirm that induction of ferroptosis markers by OGM were due to GPR68 inhibition, we assessed ferroptosis markers after *GPR68* gene knockdown in U87 and U138 GBM cells. Consistent with OGM treatment, siRNA-mediated knock-down of *GPR68* significantly increased the expression of the ferroptosis markers (*TFRC and ATF4*,), their transcriptional targets (*CHAC1 and SLC7A11)*, as well as *HMOX1*, a marker of oxidative stress Fig. S13A, C). Similar results were obtained with CRISPRi-mediated knock-down of *GPR68* (Fig. S13B, D). Congruent with the transcriptional changes observed, OGM-treatment significantly increased the protein levels of TFRC (Transferrin Receptor) and HO-1 (Heme oxygenase-1), encoded by *HMOX1* (Fig. S14A-F) [74]. Notably, OGM did not induce apoptosis, as assessed by cleaved caspase 3 levels (Fig. S14G-J).

**Fig. 6:**
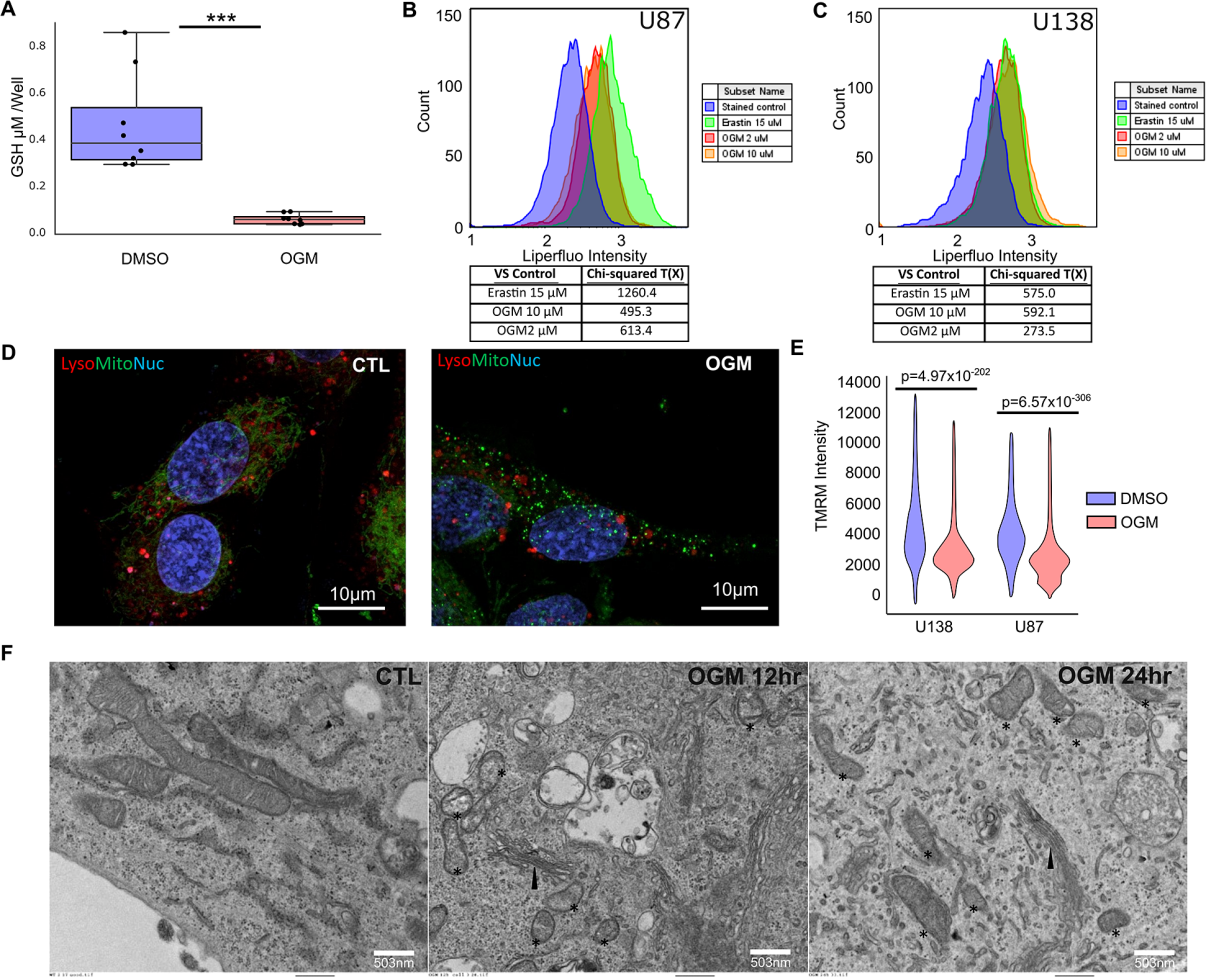
Loss of GPR68 causes ferroptosis in GBM. (**A**) OGM decreased GSH levels in U87 cells. (**B**, **C**) OGM significantly increased lipid peroxidation in U87 and U138. Chi square T(X) > 4 is considered significant. (**D**) OGM disrupted Mitochondria structure (Mitotracker) and function (TMRM) in U87, but not lysosome structure (Lysotracker). (**E**) Quantification of TMRM in U87 and U138. (**F**) Ultrastructure of U87 cells treated with OGM show disrupted mitochondria: *, but normal ER (arrowhead)

To better understand the mechanisms occurring in the cell after treatment with OGM, we investigated other hallmarks of ferroptosis. Consistent with the elevated CHAC1 levels seen in PDX, U87, and U138 cells (Fig. 5C, H; Fig. S13A-D), OGM treatment dramatically reduced glutathione levels in U87 cells (Fig. 6A) and significantly increased lipid peroxidation in U87 and U138 cells (Chi-squared T(X) > 4 is equal to *p* < 0.01) (Fig. 6B, C). In contrast to OGM treatment and GPR68 knockdown in GBM (Fig. S15A, B), OGM did not induce lipid peroxidation in HEK293 cells, consistent with the lack of effect on HEK293 survival (Fig. S15C, D). The small molecule Erastin, a classic ferroptosis inducer that inhibits the cystine-glutamate antiporter system Xc, caused both lipid peroxidation and ferroptosis in HEK293 cells (Fig. S15) [52, 75]. Thus, in contrast to Erastin, ferroptosis induction by OGM is selective for GBM cells.

Lipid peroxidation associated with ferroptosis is known to disrupt mitochondrial membranes, resulting in smaller mitochondria [74]. OGM-treated U87 cells exhibited punctate mitochondria, when stained with vital mitochondrial stain MitoTracker, with decreased mitochondrial membrane potential, as measured by TMRM (Tetramethylrhodamine, methyl ester) staining, without discernible effect on lysosomes (Fig. 6D, E). Transmission electron microscopy (TEM) of U87 cells after 12- and 24-hours of OGM treatment demonstrated smaller mitochondria with an increased incidence of ruptured membranes (Fig. 6F). Notably, OGM-treated U87 cells did not exhibit distended ER seen in the ER stress response, nor did we observe membrane blebbing seen in apoptosis (Fig. 6F). Lastly, consistent with the known synergy between other small molecule ferroptosis inducers and ionizing radiation, OGM and ionizing radiation demonstrated exceptionally strong synergistic induction of lipid peroxidation in U87 and U138 cells with a coefficient of drug interaction (CDI) < 0.06 (CDI < 1 indicates synergism; Fig. S16A-F) [76, 77]. These results further strengthen the notion that OGM induces GBM cell death via ferroptosis.

### GPR68 inhibition induces ferroptosis via an ATF4-CHAC1-dependent mechanism

**Fig. 7:**
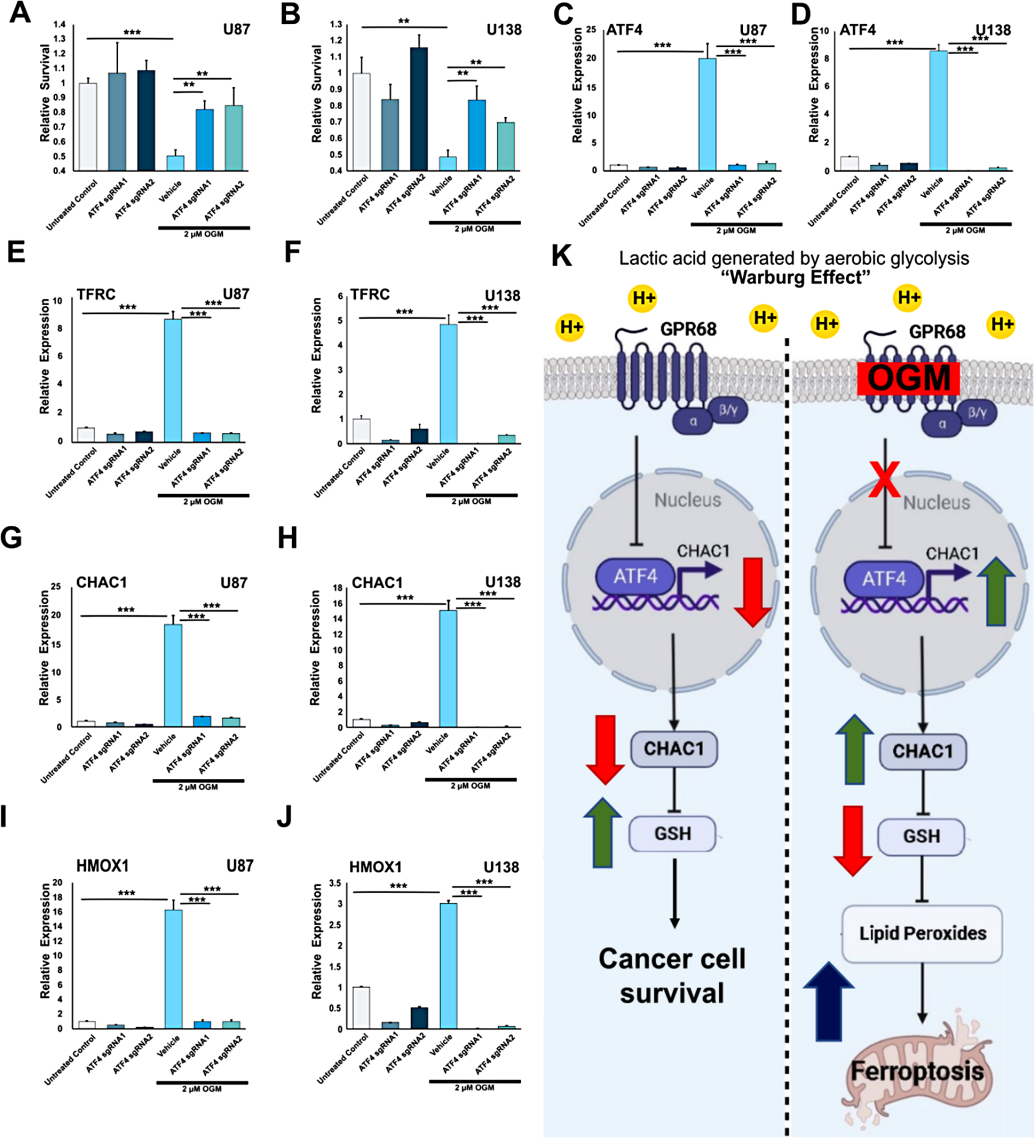
OGM induces ferroptosis through upregulation of ATF4. (**A**) CRISPRi knock-down of ATF4 prevented OGM-induced cell death in U87 cells, while guide RNAs or dCas9 alone had no effect on survival. (**B**) Knock-down of ATF4 prevented OGM-induced cell death in U138 cells. (**C**) CRISPRi successfully reduced ATF4 expression even in the setting of OGM-induced expression in U87 cells and (**D**) in U138 cells. (**E**) CRISPRi mediated knock-down of ATF4 prevented OGM-induced expression of ferroptosis marker TFRC in U87 cells and (**F**) in U138 cells. (**G**) CRISPRi knock-down of ATF4 prevented OGM induced expression of direct ATF4 target CHAC1 in U87 cells and (**H**) in U138 cells. (**I**) CRISPRi knockdown of ATF4 prevented OGM-induced increase in oxidative stress response marker HMOX1 in U87 cells and (**J**) in U138 cell. (**K**) Model depicting effects of OGM in GBM cells. Lactic acid accumulation from the Warburg effect activates GPR68, which suppresses ATF4 transcription. GPR68 inhibition by OGM induces ATF4 expression, which then increases CHAC1, leading to depletion of glutathione. This ultimately causes accumulation of toxic lipid peroxides, which triggers ferroptosis

Given that loss of GPR68 activity increased ATF4 expression, we sought to confirm whether ATF4 was required for ferroptosis induction by OGM [69, 72, 78, 79]. Knockdown of *ATF4* significantly attenuated much of the effects of OGM on U87 and U138 cells, including the induction of cell death (Fig. 7A-D; Fig. S17) as well as the ferroptosis markers *TFRC* (Fig. 7E, F; S18A, B), *CHAC1* (Fig. 7G, H; S18C, D), and *SLC7A11* (Fig. S18E, F), and the oxidative stress marker *HMOX1* (Fig. 7I, J, S18G, H) [80–82]. By comparison, negative controls (Cas9 alone and sgRNAs alone) had no effect on any of these genes (Fig. S19). These results suggest that inhibition of the extracellular acid-induced signaling by GPR68 induces ferroptosis via ATF4.

To confirm whether OGM-mediated induction of ATF4 activity is sufficient to induce ferroptosis, we sought to investigate CHAC1, a direct target of the ATF4 transcription factor [69, 71, 72, 78, 83]. Congruent with RNA expression data, OGM elevated both ATF4 and CHAC1 protein levels (Fig. S20A-F). Furthermore, ATF4 overexpression alone proved sufficient to induce CHAC1 expression in GBM cells (Fig. S20A-F), resulting in the generation of oxidized lipids (Fig. S20G, I) and subsequent cell death in glioblastoma cells (Fig. S20H, J). While ATF4 is known to govern expression of many genes, knockdown of *CHAC1* also significantly attenuated much of the OGM-induced cell death of both U87 and U138 cells (Fig. S21A, B). Consistent with ATF4 being upstream of CHAC1, knockdown of *CHAC1* did not impact the levels of ATF4 in OGM treated GBM cells (Fig. S21C, D). Importantly, this discrepancy was not attributable to inadequate *CHAC1* knockdown (Fig. S21E, F). Consistent with the role of CHAC1 as a negative regulator of glutathione, *CHAC1* knockdown attenuated OGM induction of the oxidative stress marker *HMOX1* (Fig. S20G, H) and the ferroptosis markers *TFRC* (Fig. S20I, J). In summary, OGM’s inhibition of GPR68 leads to increased ATF4 expression, which in turn drives the transcription of CHAC1, a well-known GSH degrader. This cascade results in the depletion of GSH, subsequently precipitating lipid peroxidation and ultimately triggering ferroptosis (Fig. 7K).

## Discussion

We report the identification of a novel class of small molecules, which we named ogremorphins (OGMs), that specifically antagonize GPR68, an extracellular proton-sensing GPCR. Using this drug class and genetic means, we demonstrate that GPR68 mediates a critical pro-survival pathway activated in glioblastoma cells in an autocrine manner by the acidic extracellular milieu. The acidic tumor microenvironment, generated in large part by the Warburg effect, is a common feature of solid cancers thought to play an important role in tumor progression, metastasis, immune evasion, and other pro-oncogenic behaviors [7–14]. Recent findings have implicated GPR68 in the pro-oncogenic effects of the TME, with its activity in cancer-associated fibroblasts being critical for tumor growth in pancreatic cancer [21]. Moreover, clinical evidence indicate that the patients using anxiolytic Lorazepam, which has off-target agonism of GPR68, had a 3.8-fold higher rate of recurrent pancreatic cancer compared to control group [23, 24]. This finding also was not limited to pancreatic cancer, but showed that the lorazepam use was correlated with significantly worse overall survival in prostate, ovarian, head and neck, uterine, colon, and breast cancer, and melanoma [23, 24]. However, a direct role of GPR68 in tumor cells remained unexplored. Here, we demonstrate that GBM cells, expressing GPR68, respond to media acidification with a Ca2 + response, which was sensitive to the GPR68 inhibitor ogremorphin (OGM) and a PLC inhibitor, indicating that GPR68/Gq mediated extracellular acid signaling. Notably, this response mirrors findings in medulloblastoma cells, suggesting a shared mechanism for acidic TME response in these two CNS tumor types [16].

Mechanistically, our investigation reveals that GPR68 inhibition induces ferroptosis in GBM cells through the upregulation of ATF4 and its downstream target, CHAC1. CHAC1, a direct transcriptional target of ATF4, induces oxidative stress by degrading glutathione (GSH), leading to increased lipid peroxidation and mitochondria disintegration—hallmarks of ferroptosis. While RNA-seq results suggest OGM induces both ferroptosis and the unfolded protein response/endoplasmic reticulum (UPR/ER) stress response, electron microscopy studies did not reveal evidence of ER stress response. ATF4, implicated in ferroptotic neuronal death during stroke, demonstrated a similar induction of ferroptosis in GBM cells [84]. GPR68, known to protect neurons from death in ischemic stroke, suggests a potential mechanism for cancer cells to resist oxidative stress [85]. Elevated reactive oxygen species in many cancers, including GBM, trigger metabolic reprogramming, contributing to TME acidification. Our data support the hypothesis that GPR68, by repressing ATF4 and CHAC1, serves as a protective mechanism against oxidative stress in the context of acidic TME downstream of the Warburg Effect.

Many of the current anti-cancer therapies aim to induce apoptosis. However, this process depends on the p53 tumor suppressor, which is dysfunctional in vast majority of GBM cases [86]. Recently, ferroptosis has emerged as an intriguing alternative cell death pathway for cancer treatment [86–91]. Based on our findings, we propose that small molecule GPR68 inhibitors like OGM, which selectively induce ferroptosis in GBM cells, represents a promising therapeutic avenue for this deadly cancer. Moreover, consistent with earlier observations that low extracellular pH confers radio-resistance and GPR68 is upregulated in radioresistant cell lines, [18, 19] OGM demonstrated a synergistic induction of ferroptosis in GBM cells. OGM also demonstrated synergistic GBM cell killing with TMZ. Importantly, since the GPR68-mediated pro-survival mechanism is activated only in the setting of the acidic milieu of cancers, noncancerous tissues should not be affected, as evidenced by the lack of cell death caused by OGM in fibroblasts and normal neural tissue. In summary, our findings underscore the critical role of GPR68 in the autocrine interplay between the Warburg Effect, acidic TME, and protection from ferroptosis in GBM cells, suggesting that GPR68 inhibitors like ogremorphins may offer an appealing therapeutic strategy, especially in combination with the frontline therapies such as TMZ and ionizing radiation.

### Electronic supplementary material

Below is the link to the electronic supplementary material.

Supplementary Material 1

Supplementary Material 2

Supplementary Material 3

## Data Availability

The datasets used and/or analyzed during the current study are available from the corresponding author on reasonable request.
