# Supplementary material for: GPR68-ATF4 signaling is a novel prosurvival pathway in glioblastoma activated by acidic extracellular microenvironment
Source: Exp Hematol Oncol. 2024 Jan 31;13:13. doi: 10.1186/s40164-023-00468-1 (PMC10829393; doi:10.1186/s40164-023-00468-1)
Supplement: Supplementary file 1 — Supplementary Material 1 [file 40164_2023_468_MOESM1_ESM.docx]

**Supplementary Figure and Table Legends**

**Figure S1.** **OGM1 affects pigment development in zebrafish embryo.** **(A)** Craniofacial cartilage stained with Alcian blue viewed laterally and ventrally, pigmentation and notochord viewed laterally. Ogremorphin treatment resulted in craniofacial dysmorphogenesis, disrupted pigmentation, and a wavy notochord (Red arrow), (quantification of phenotypes is provided in Extended Data 5). **(B)** Consistent with ogremorphin treatment, Morpholino and Cas9 knockdown of GPR68 resulted in craniofacial dysmorphogenesis, disrupted pigmentation, and a wavy notochord (Red arrow) (quantification of phenotypes is provided in Extended Data 5). Cas9^GPR68^ = GPR68 targeted sgRNA and Cas9; CTL= control; MoGPR68 = GPR68 morpholino treatment.

**FigureS2.** **Quantification of GPR68 knockdown and inhibition phenotypes.** Morpholino-injected embryos exhibited dose-dependent increases in phenotype prevalence with a shortened body axis, abnormal pigmentation, and defects in notochord defects at 1.5 ng and 3 ng. The mismatched morpholino (3 ng) had minimal effects. OGM had increasing effects on these phenotypes at 10 and 20 μM. Cas9 targeting of GPR68 generated the same phenotype as both OGM treatment and GPR68 knockdown with a shortened body axis, abnormal pigmentation, and defects in notochord integrity. MM = mismatch morpholino; MO = morpholino.

**Figure S3. OGM resynthesis route.**

**Figure S4. Characterization of OGM resynthesis. (A)** Proton nuclear magnetic resonance of OGM resynthesis. **(B)** Liquid chromatography/mass spectrometry of OGM resynthesis*.*

**Figure S5.** **Similarity and identity between** **proton-sensing GPCRs. (A)** Identity and similarity between human and zebrafish orthologs. **(B)** Similarity of acid-sensing GPCRs in zebrafish.

**Figure S6.** **Glioma spheroids form organized highly acidotic cores not dependent on size**. **(A)** Representative images of spheroids made with U87 cells expressing pHluorin2-GPI, acquired over time from different seeding densities **(B)** Quantification of mean intensity of spheroids shown in (A) with 95% CI. The extracellular environment of glioblastoma spheroids shows progressive acidification detected via an increase in 469ex/525em of extracellular pH indicator pHluorin2-GPI (n=12 spheroids per condition).

**Figure S7.** **OGM synergizes with the frontline therapeutic Temozolomide. (A)** Dose response of OGM on PDX 08-387 glioblastoma cell survival. **(B)** Concurrent treatment of PDX with OGM and TMZ at increasing concentrations of TMZ. **(C)** Calculated coefficient of drug interaction (CDI) of <0.7, indicated strong synergistic killing by OGM and TMZ.

**Figure S8. OGM reduces viability of Human PDX and Mouse Glioblastoma lines through GPR68. (A, B)** CRISPRi targeting GPR68 in U87 cells, with additional guides against GPR68 reduced both survival and expression of GPR68, while sgRNA alone and dCas9 alone have no effect on survival or expression. **(C, D)** CRISPRi targeting GPR68 in U138 cells, with additional guides against GPR68 reduced both survival and expression of GPR68, while sgRNA alone and dCas9 alone have no effect on survival or **expression. (E)** OGM reduced the viability of 2 PDX lines, and a mouse model of glioblastoma in 2D cell survival assays. **(F)** OGM reduced the viability of 2 PDX lines in 3D tumor spheroid assays.

**Figure S9. OGM potently inhibits survival of diverse glioblastoma cell lines.** Summary of LC_50_ of OGM for 13 GBM cell lines, showing efficacy of OGM across diverse genetic backgrounds, both sexes and two species.

**Figure S10.** **OGM causes specific cell death in glioblastoma cells.** **(A)** OGM reduced U87 viability, but not HEK293 cell. (**B**) Quantification of cell survival assay. **(C)** Acridine orange stain of zebrafish embryos treated with OGM to visualize cell death. (**D**) Quantification of results showed OGM did not cause significant excess death in zebrafish embryos. **(E)** Tg(NeuroD1:EGFP) zebrafish treated at 18hpf, with OGM (5µM) showed no significant increase in cell death in neurons and glial cells. **(F)** Quantification of **(E)** (N=20 embryos per condition).

**Figure S11. Acid induced GPR68 activation promotes cell survival in glioblastoma.**

**(A)** Acidiic media (pH 6.2) promoted U87 spheroid growth at 3 days, in comparison to the basic media (pH 8,0; P<0.0001). The enhanced growth in acidic media was blocked by OGM (P<0.0001) (N=8 for each condition). **(B)** Quantification of (A) (N=8 for each condition). **(C)** Growth trends of U87 spheroids treated with Ogerin (OGRN), OGM and DMSO vehicle. OGRN promoted U87 spheroids growth rate, whereas OGM impaired spheroid growth rate. N=8 for each condition. **(D)** OGM, caused dose-dependent inhibition of U87 cell growth in 2D culture in both pH6.2 and pH7.8. **(E)** OGM, caused dose-dependent inhibition of U138 cell growth in 2D culture in both pH6.2 and pH7.8.

**Figure S12. Gene set enrichment analysis of share dysregulated pathways.** Gene set enrichment analysis applied to significantly differentially expressed genes (SDEG) from each cell type. Significantly enriched terms shared by 2 or more cell types are shown. (Subset of analysis in Figure 5G).

**Figure S13. Knockdown of GPR68 recapitulates OGM induced ferroptosis gene signature seen in PDX cells (A)** siRNA knock-down of GPR68 increased expression of the ferroptosis markers TFRC, ATF4, and CHAC1, and the oxidative stress marker HMOX1 in U87 cells, like the OGM treatment, whereas control siRNA had no effect. **(B)** CRISPRi knock-down of GPR68 increased expression of TFRC, ATF4, CHAC1, and HMOX1 in U87 cells, while guide RNAs or dCas9 alone had no effect.  **(C)** siRNA knock down of GPR68 increased expression of TFRC, ATF4, CHAC1, and HMOX1 in U138 cells. **(D)** CRISPRi knock-down of GPR68 increased expression of TFRC, ATF4, CHAC1, and HMOX1 in U138 cells.

**Figure S14. OGM induces ferroptosis but not apoptosis.** **(A-C)** OGM increased protein expression of TFRC, a marker of ferroptosis and increased protein expression of HO-1, a marker of oxidative stress in U87 cells **(D-F)** OGM increased protein expression of TFRC, a marker of ferroptosis and increased protein expression of HO-1, a marker of oxidative stress in U138 cells **(G-J)** OGM did not increase cleaved caspase 3 level, whereas positive control doxorubicin does in U87 cells **(G-H)** and U138 **(I-J)**. (All quantifications are from 3 biological replicates.)

**Figure S15 Loss of GPR68 activity specifically induces ferroptosis in GBM cells.**

**(A)** Knock down of GPR68 increased lipid peroxidation in U87 and U138 cells. **(B)** Knockdown of GPR68 was highly significant with Chi-squared >4, which is equal to p<0.01. **(C)** Erastin but not OGM, increased lipid peroxidation in HEK293. **(D)** Erastin, but not OGM, dramatically reduced HEK293 cell survival.

**Figure S16. OGM synergizes with radiation. (A-C)** OGM and 2Gy ionizing radiation demonstrated very strong synergy (CDI <0.06) for inducing lipid peroxidation in U87 cells. All treatments were highly significant with Chi-squared >4, which is equal to p<0.01) **(D-F)** OGM and 2Gy ionizing radiation demonstrated exceptionally strong synergy (CDI <0.006) for inducing lipid peroxidation in U138 cells. All treatments were highly significant with Chi-squared >4, which is equal to p<0.01)

**Figure S17. OGM induces ferroptosis through upregulation of ATF4. (A)** CRISPRi knock-down of ATF4, with additional sgRNAs, prevented OGM-induced cell death in U87 and U138 cells, while guide RNAs or dCas9 alone had no effect on survival. **(B)** Additional ATF4 targeting CRISPRi successfully reduced ATF4 expression even in the setting of OGM-induced expression in U87 cells and in U138 cells.

**Figure S18. ATF4 knockdown prevents transcription of Ferroptosis Indicators. (A)** CRISPRi knock-down prevented OGM-induced expression of ferroptosis marker TFRC in U87 cells and **(B)** in U138 cells. **(C)** CRISPRi knock-down of ATF4 prevented OGM induced expression of direct ATF4 target CHAC1 in U87 cells and **(D)** in U138 cells. **(E)** CRISPRi knock-down of ATF4 prevented OGM induced expression of direct ATF4 target SLC7A11 in U87 cells and **(F)** in U138 cells. **(G)** CRISPRi knockdown of ATF4 prevented OGM-induced increase in oxidative stress response marker HMOX1 in U87 cells and **(H)** in U138 cell.

**Figure S19. ATF4 targeting guides alone and Cas9 alone have no effect on ferroptosis markers. (A)** Controls for ATF4 CRISPRi knock-down had no effect on the expression of ferroptosis marker TFRC in U87 cells and **(B)** in U138 cells. **(C)** Controls for ATF4 CRISPRi knock-down had no effect on the expression of direct ATF4 target CHAC1 in U87 cells and **(D)** in U138 cells. **(E)** Controls for ATF4 CRISPRi knock-down had no effect on the expression of direct ATF4 target SLC7A11 in U87 cells and **(F)** in U138 cells. **(G)** Controls for ATF4 CRISPRi knock-down had no effect on expression of oxidative stress response marker HMOX1 in U87 cells and **(H)** in U138 cell.

**Figure S20. ATF4 expression is sufficient for inducing ferroptosis.** Treatment with OGM induced ATF4 and CHAC1 protein expression in U87 **(A-C)** and U138 cells **(D-F)** (All quantifications are from 3 biological replicates). Over-expression of ATF4 was sufficient to induce CHAC1 protein overexpression **(A-F)**. ATF4 over-expression increases lipid peroxidation cells and significantly decreases survival of U87 **(G-H)** and U138 cells **(I-J)** (n=3).

**Figure S21. OGM induces ferroptosis through upregulation of CHAC1. (A)** CRISPRi knock-down off CHAC1, prevented OGM-induced cell death in U87, while guide RNAs or dCas9 alone had no effect on survival. **(B)** CRISPRi knock-down off CHAC1, prevented OGM-induced cell death in U138, while guide RNAs or dCas9 alone had no effect on survival. **(C)** CRISPRi knock-down had no effect on OGM-induced expression of ATF4 in U87 cells and **(D)** in U138 cells. **(E)** CRISPRi knock-down of CHAC1 prevented OGM induced expression of direct ATF4 target CHAC1 in U87 cells and **(F)** in U138 cells. **(G)** CRISPRi knockdown of CHAC1 prevented OGM-induced increase in oxidative stress response marker HMOX1 in U87 cells and **(H)** in U138 cell. **(I)** CRISPRi knock-down prevented OGM-induced expression of ferroptosis marker TFRC in U87 cells and **(J)** in U138 cells.

**Figure S22. Expression of GPR68 in GBM lines** Compared to OGM insensitive HEK293, U87, U138, Mayo6, Mayo39 and 08-387 lines had significantly increased expression of GPR68.

**Supplemental Tables**

**Supplemental Table 1. KinomeScan (DiscoverRx) profiling of OGM1**

**Supplemental Table 2. GPCR Profiler (Millipore) profiling of OGM1**

**Supplemental Table 3. Normalized reads from RNA-seq of OGM treated GBM cells**

**Supplemental Table 4. Differentially expressed (FDR <0.01) genes in Mayo Cells**

**Supplemental Table 5. Differentially expressed (FDR <0.01) genes in 913 Cells**

**Supplemental Table 6. Differentially expressed (FDR <0.01) genes in 08-387 Cells**

**Supplemental Table 7. 7 Shared differentially expressed genes**

**Supplemental Table 8. Shared terms from Gene Set enrichment Analysis differentially expressed genes across cell lines**
