# Supplementary material for: GPR68-ATF4 signaling is a novel prosurvival pathway in glioblastoma activated by acidic extracellular microenvironment
Source: Exp Hematol Oncol. 2024 Jan 31;13:13. doi: 10.1186/s40164-023-00468-1 (PMC10829393; doi:10.1186/s40164-023-00468-1)
Supplement: Supplementary file 2 — Supplementary Material 2 [file 40164_2023_468_MOESM2_ESM.pdf]

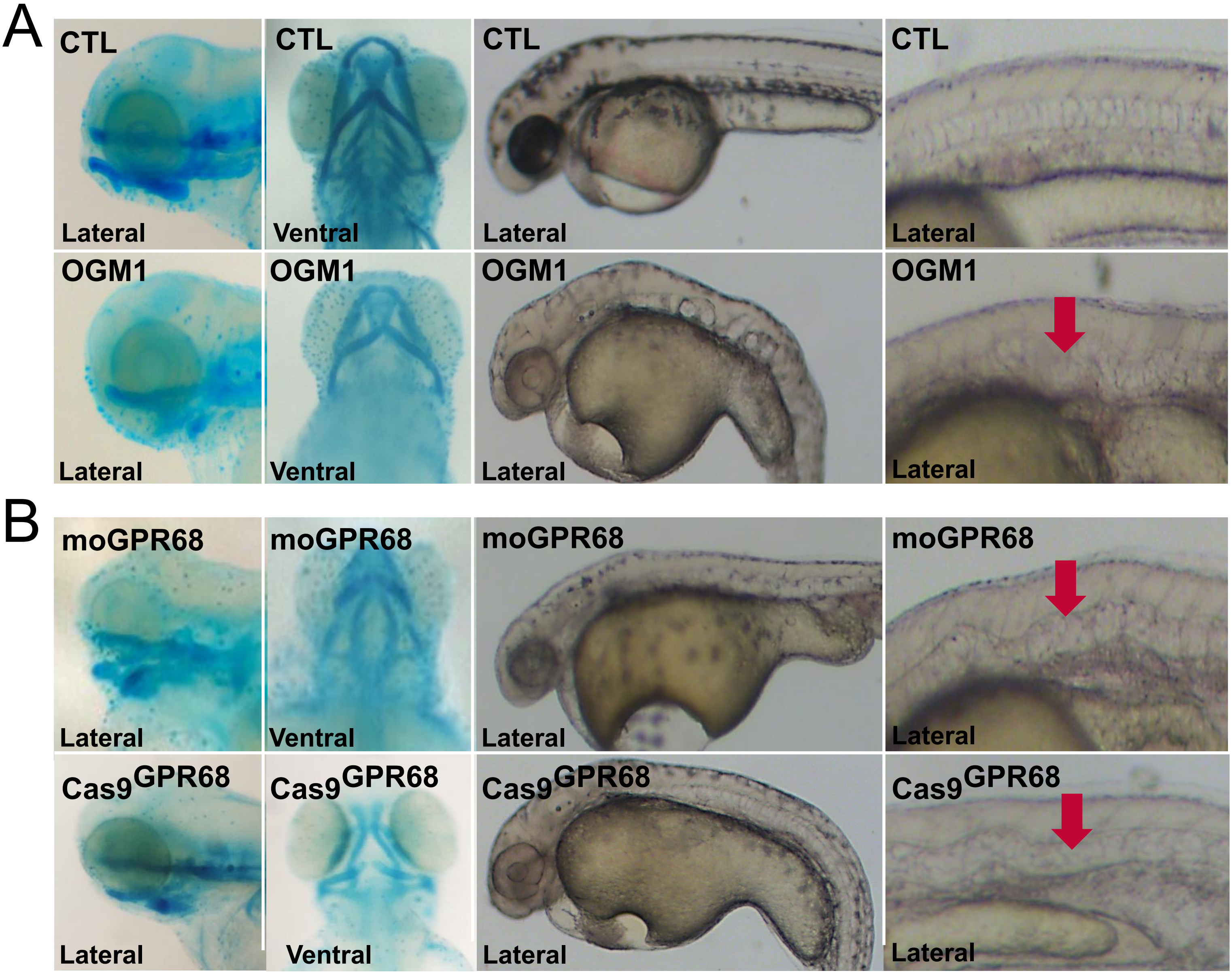
A
CTL
Lateral
CTL
Ventral
CTL
Lateral
CTL
Lateral
OGM1
Lateral
OGM1
Ventral
OGM1
Lateral
OGM1
Lateral
B
moGPR68
Lateral
moGPR68
Ventral
moGPR68
Lateral
moGPR68
Lateral
Cas9GPR68
Lateral
Cas9GPR68
Ventral
Cas9GPR68
Lateral
Cas9GPR68
Lateral

Figure S1

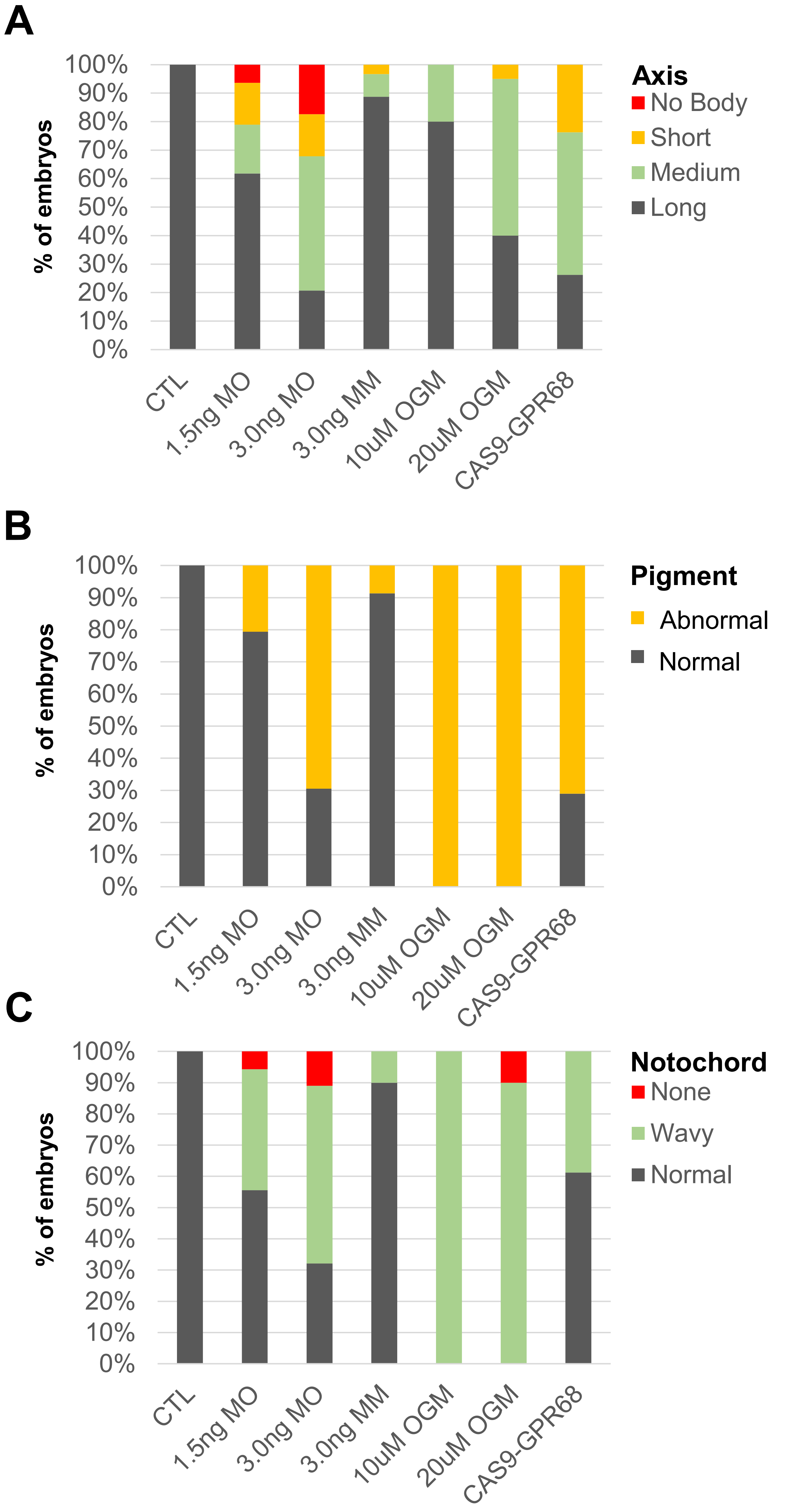
A
100%
90%
80%
70%
60%
50%
40%
30%
20%
10%
0%
% of embryos
CTL
1.5ng MO
3.0ng MO
3.0ng MM
10uM OGM
20uM OGM
CAS9-GPR68
Axis
No Body
Short
Medium
Long
B
% of embryos
Pigment
Abnormal
Normal
C
% of embryos
Notochord
None
Wavy
Normal

Figure S2

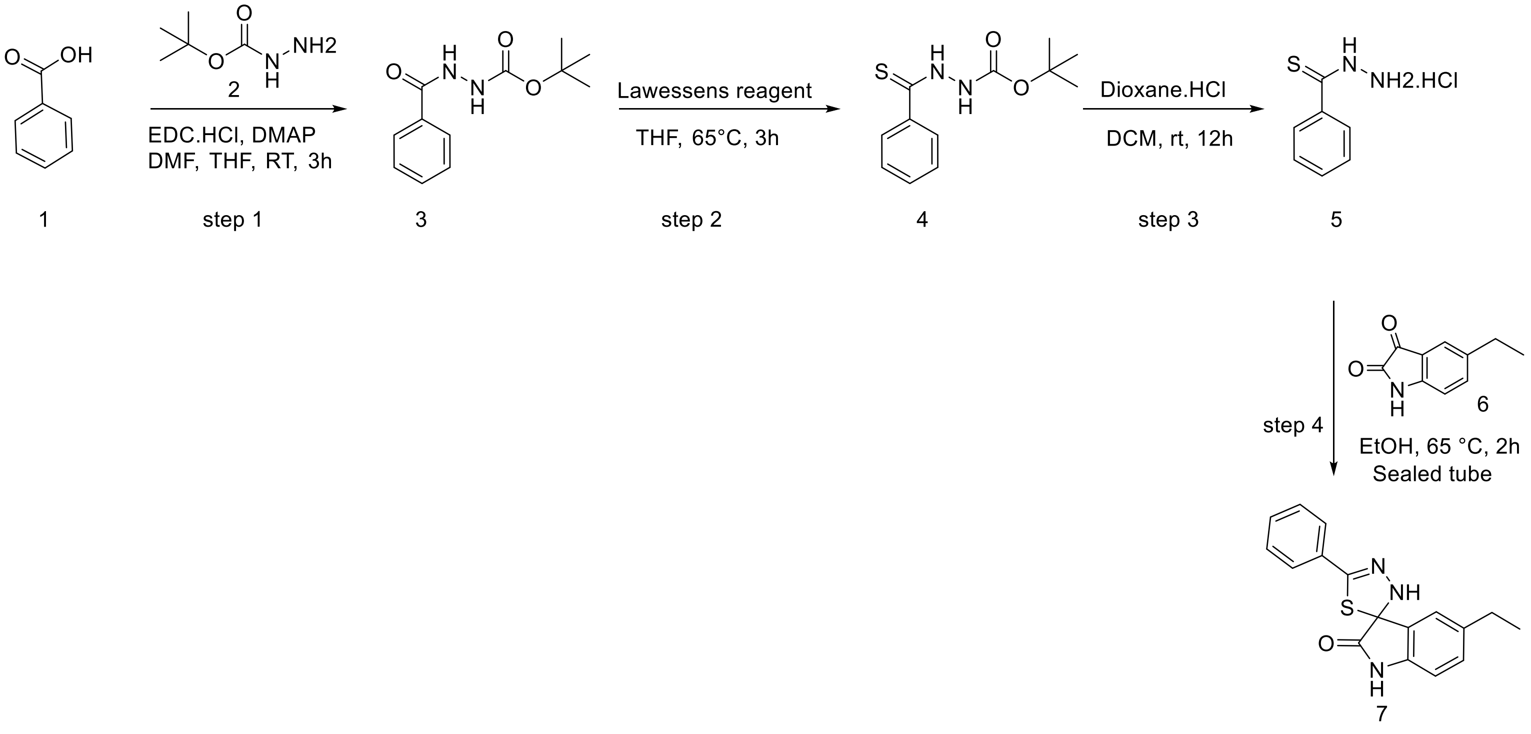

2
EDC.HCl, DMAP
DMF, THF, RT, 3h
step 1
Lawessens reagent
THF, 65°C, 3h
step 2
Dioxane.HCl
DCM, rt, 12h
step 3
NH2.HCl
6
step 4
EtOH, 65 °C, 2h
Sealed tube
1
3
4
5
7

Figure S3

A

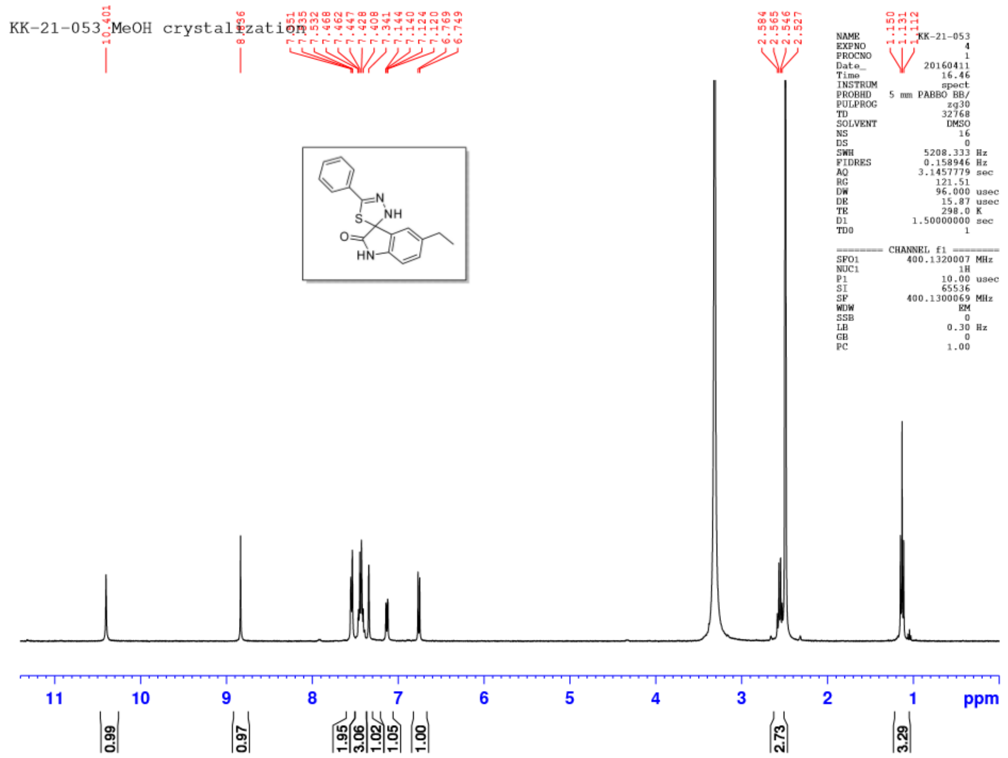

B

Peak #	Time	Area % UV215	Area % UV254	Area % TIC(+)	BPM	Area Abs UV215	Area Abs UV254	Area Abs TIC(+)
1	0.241	37.6	0.0	5.2	157.2	658.96	0	81116.66
2	0.349	0.0	0.0	9.3	159.2	0	0	144753.36
3	1.200	62.4	100.0	85.5	310.1	1093.91	580.14	1333471.38

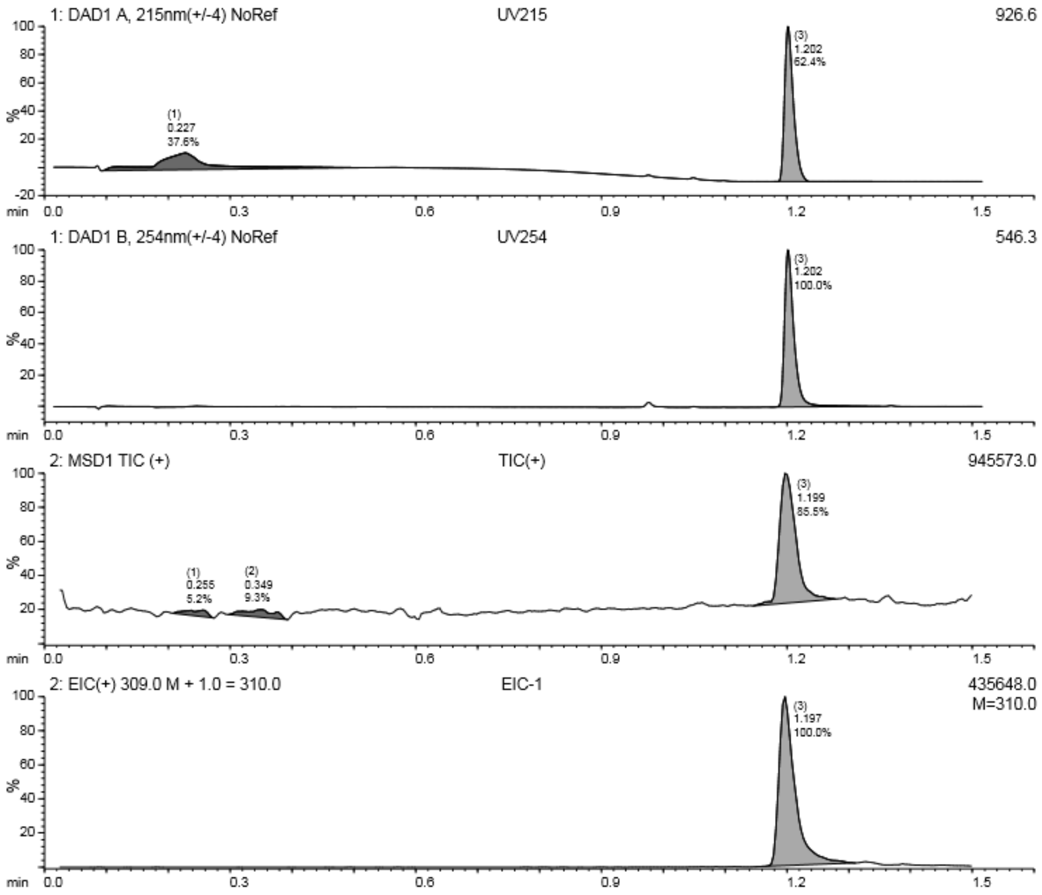

Figure S4

A

	Identity % (AA)	Similarity % (AA)
**Hs. GPR65 vs. Dr.GPR65**	39	58.8
**Hs. GPR68 vs. Dr.GPR68**	58.3	75.2
**Hs. GPR4 vs. Dr.GPR4**	74.1	85.2

B

	Dr.GPR65	Dr.GPR68	Dr.GPR4
Dr.GPR65	100	28.04	30.61
Dr.GPR68	28.04	100	41.45
Dr.GPR4	30.61	41.45	100

Figure S5

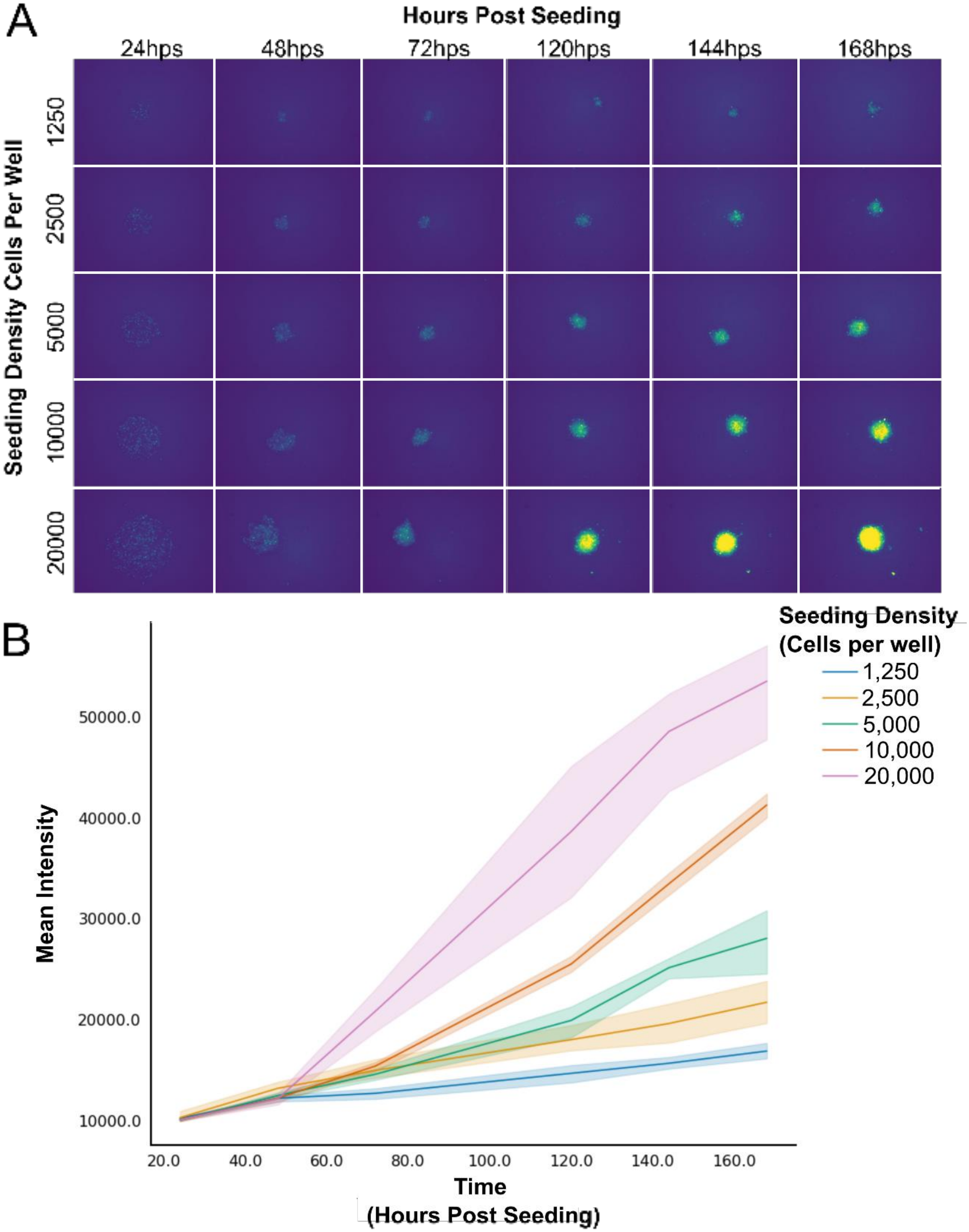
A
Hours Post Seeding
24hps
48hps
72hps
120hps
144hps
168hps
Seeding Density Cells Per Well
1250
2500
5000
10000
20000
B
Mean Intensity
50000.0
40000.0
30000.0
20000.0
10000.0
20.0
40.0
60.0
80.0
100.0
120.0
140.0
160.0
Time
(Hours Post Seeding)
Seeding Density
(Cells per well)
1,250
2,500
5,000
10,000
20,000

Figure S6

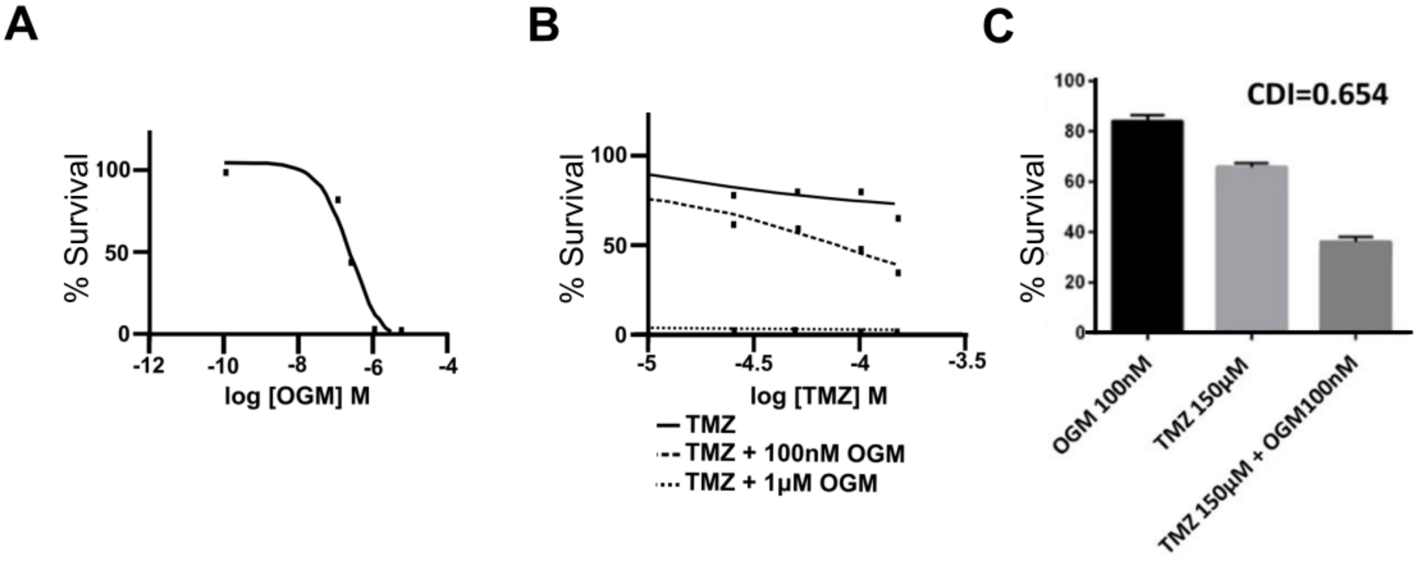
A
% Survival
100
50
0
-12
-10
-8
-6
-4
log [OGM] M
B
% Survival
100
50
0
-5
-4.5
-4
-3.5
log [TMZ] M
TMZ
TMZ + 100nM OGM
TMZ + 1μM OGM
C
CDI=0.654
% Survival
100
80
60
40
20
0
OGM 100nM
TMZ 150μM
TMZ 150μM + OGM100nM

Figure S7

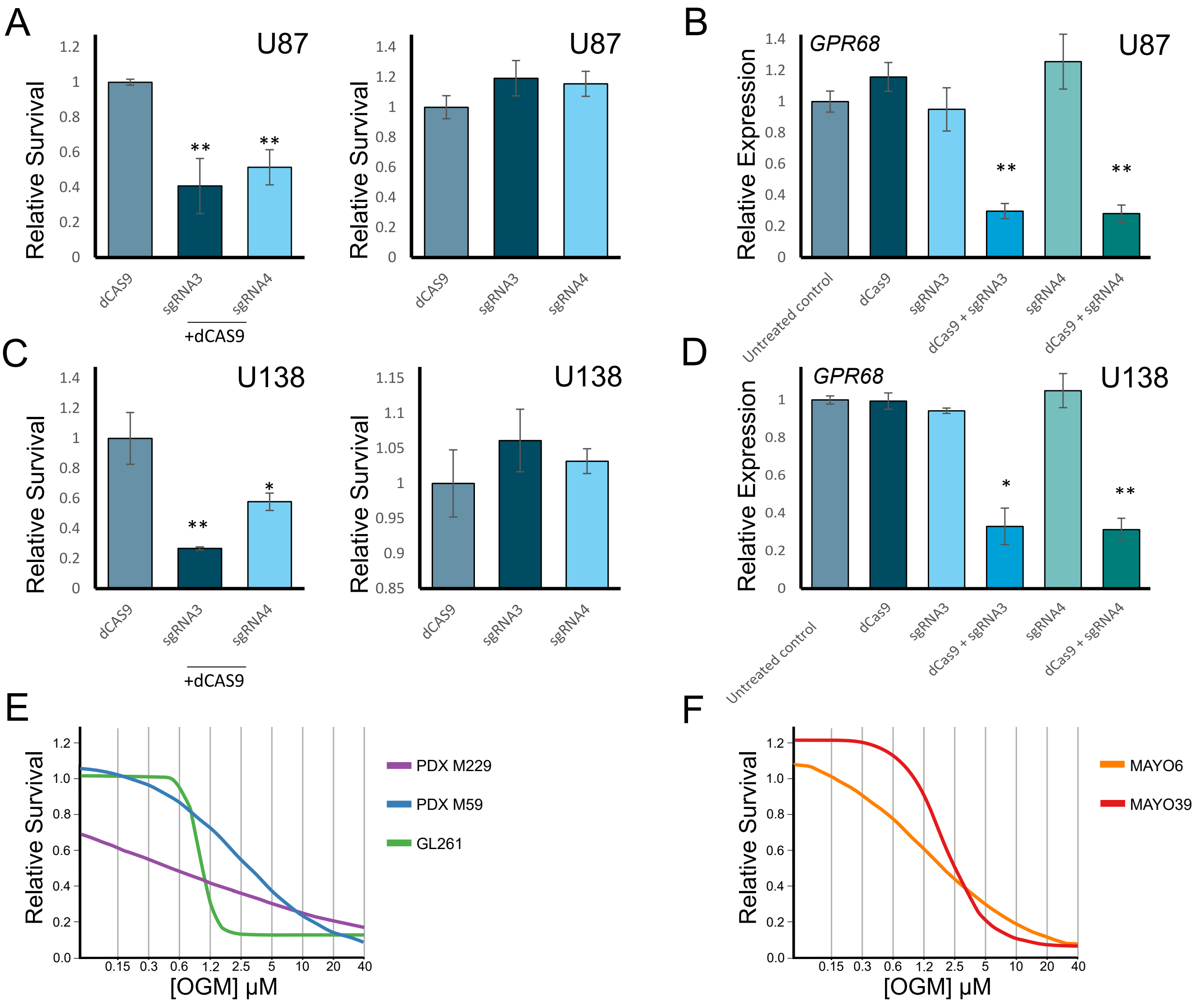
A
U87
Relative Survival
dCAS9
sgRNA3
sgRNA4
+dCAS9
**
**
U87
Relative Survival
dCAS9
sgRNA3
sgRNA4
B
GPR68
U87
Relative Expression
Untreated control
dCas9
sgRNA3
dCas9 + sgRNA3
sgRNA4
dCas9 + sgRNA4
C
U138
Relative Survival
dCAS9
sgRNA3
sgRNA4
+dCAS9
U138
Relative Survival
dCAS9
sgRNA3
sgRNA4
D
GPR68
U138
Relative Expression
Untreated control
dCas9
sgRNA3
dCas9 + sgRNA3
sgRNA4
dCas9 + sgRNA4
E
Relative Survival
[OGM] µM
PDX M229
PDX M59
GL261
F
Relative Survival
[OGM] µM
MAYO6
MAYO39

Figure S8

GBM Cells	Species	Sex	TMZ sensitivity	Molecular subtype	EGFR amplification	EGFR Mutation	TP53	LC_{50} (uM)
U87	Human	M	Sensitive (1)	Mesenchymal	WT (2)	WT (2)	WT (3)	0.75
U138	Human	M	Resistant (4)	Mesenchymal	WT(5)	WT(5)	MT (6)	0.92
913 (7)	Human	M	Resistant (8)	UNK	Amplified (9)	WT (9)	MT (10)	0.45
08-387	Human	UNK	Resistant (11)	UNK	UNK	UNK	UNK	0.42
3691	Human	UNK	UNK	UNK	UNK	UNK	UNK	0.43
1079	Human	UNK	UNK	UNK	UNK	UNK	UNK	1.99
PDX-M6 (12)	Human	M	Resistant (12)	Classical (12)	Amplified (12)	VIII (12)	WT (12)	2.7
PDZ-M28	Human	M	Resistant (12)	UNK (12)	WT (12)	WT (12)	LOSS (12)	0.73
PDX-M39	Human	M	Sensitive (12)	Mesenchymal (12)	Amplified (12)	VIII (12)	WT (12)	0.49
PDX-M59	Human	F	Sensitive (12)	Mesenchymal (12)	Amplified (12)	VIII (12)	LOSS (12)	0.55
PDX-M229	Human	F	Resistant (12)	UNK (12)	WT (12)	WT (12)	WT (12)	0.43
KT158-Luc	Mouse	UNK	Resistant (13)	UNK	UNK	WT	UNK	0.78
GL261-Luc	Mouse	UNK	Sensitive (13)	UNK	UNK	WT	UNK	0.39

(1) Baer JC, Freeman AA, Newlands ES, Watson AJ, Rafferty JA, Margison GP. Depletion of O6-alkylguanine-DNA alkyltransferase correlates with potentiation of temozolomide and CCNU toxicity in human tumour cells. Br J Cancer. 1993 Jun;67(6):1299-302. doi: 10.1038/bjc.1993.241 PMID: 8512814; PMCID: PMC1968485.

(2)Liu F, Hon GC, Villa GR, Turner KM, Ikegami S, Yang H, Ye Z, Li B, Kuan S, Lee AY, Zanca C, Wei B, Lucey G, Jenkins D, Zhang W, Barr CL, Furnari FB, Cloughesy TF, Yong WH, Gahman TC, Shiau AK, Cavenee WK, Ren B, Mischel PS. EGFR Mutation Promotes Glioblastoma through Epigenome and Transcription Factor Network Remodeling. Mol Cell. 2015 Oct 15;60(2):307-18. doi: 10.1016/j.molcel.2015.09.002. Epub 2015 Oct 8. PMID: 26455392; PMCID: PMC4609298.

(3) Sesen J, Dahan P, Scotland SJ, Saland E, Dang VT, Lemarié A, Tyler BM, Brem H, Toulas C, Cohen-Jonathan Moyal E, Sarry JE, Skuli N. Metformin inhibits growth of human glioblastoma cells and enhances therapeutic response. PLoS One. 2015 Apr 13;10(4):e0123721. doi: 10.1371/journal.pone.0123721. PMID: 25867026; PMCID: PMC4395104.

(4) Grogan PT, Sarkaria JN, Timmermann BN, Cohen MS. Oxidative cytotoxic agent withaferin A resensitizes temozolomide-resistant glioblastomas via MGMT depletion and induces apoptosis through Akt/mTOR pathway inhibitory modulation. Invest New Drugs. 2014 Aug;32(4):604-17. doi: 10.1007/s10637-014-0084-7. Epub 2014 Apr 10. PMID: 24718901; PMCID: PMC4380174.

(5) Guo D, Hildebrandt IJ, Prins RM, Soto H, Mazzotta MM, Dang J, Czernin J, Shyy JY, Watson AD, Phelps M, Radu CG, Cloughesy TF, Mischel PS. The AMPK agonist AICAR inhibits the growth of EGFRvIII-expressing glioblastomas by inhibiting lipogenesis. Proc Natl Acad Sci U S A. 2009 Aug 4;106(31):12932-7. doi: 10.1073/pnas.0906606106. Epub 2009 Jul 22. PMID: 19625624; PMCID: PMC2714280.

(6)Wang X, Chen JX, Liu YH, You C, Mao Q. Mutant TP53 enhances the resistance of glioblastoma cells to temozolomide by up-regulating O(6)-methylguanine DNA-methyltransferase. Neurol Sci. 2013 Aug;34(8):1421-8. doi: 10.1007/s10072-012-1257-9 Epub 2012 Dec 8. PMID: 23224642.

(7)Galli R, Binda E, Orfanelli U, Cipelletti B, Gritti A, De Vitis S, Fiocco R, Foroni C, Dimeco F, Vescovi A. Isolation and characterization of tumorigenic, stem-like neural precursors from human glioblastoma. Cancer Res. 2004 Oct 1;64(19):7011-21. doi: 10.1158/0008-5472.CAN-04-1364. PMID: 15466194.

(8)Xia S, Lal B, Tung B, Wang S, Goodwin CR, Laterra J. Tumor microenvironment tenascin-C promotes glioblastoma invasion and negatively regulates tumor proliferation. Neuro Oncol. 2016 Apr;18(4):507-17. doi: 10.1093/neuonc/nov171 Epub 2015 Aug 27. PMID: 26320116; PMCID: PMC4799677.

(9) Gao M, Fu Y, Zhou W, Gui G, Lal B, Li Y, Xia S, Ji H, Eberhart CG, Laterra J, Ying M. EGFR Activates a TAZ-Driven Oncogenic Program in Glioblastoma. Cancer Res. 2021 Jul 1;81(13):3580-3592. doi: 10.1158/0008-5472.CAN-20-2773. Epub 2021 Apr 28. PMID: 33910930; PMCID: PMC8277712.

(10)Gampa G, Kenchappa RS, Mohammad AS, Parrish KE, Kim M, Crish JF, Luu A, West R, Hinojosa AQ, Sarkaria JN, Rosenfeld SS, Elmquist WF. Enhancing Brain Retention of a KIF11 Inhibitor Significantly Improves its Efficacy in a Mouse Model of Glioblastoma. Sci Rep. 2020 Apr 16;10(1):6524. doi: 10.1038/s41598-020-63494-7. PMID: 32300151; PMCID: PMC7162859.

(11) Tallman MM, Zalenski AA, Deighen AM, Schrock MS, Mortach S, Grubb TM, Kastury PS, Huntoon K, Summers MK, Venere M. The small molecule drug CBL0137 increases the level of DNA damage and the efficacy of radiotherapy for glioblastoma. Cancer Lett. 2021 Feb 28;499:232-242. doi: 10.1016/j.canlet.2020.11.027. Epub 2020 Nov 27. PMID: 33253788; PMCID: PMC7779703.

(12)https://www.mayo.edu/research/labs/translational-neuro-oncology/mayo-clinic-brain-tumor-patient-derived-xenograft-national-resource

Figure S9

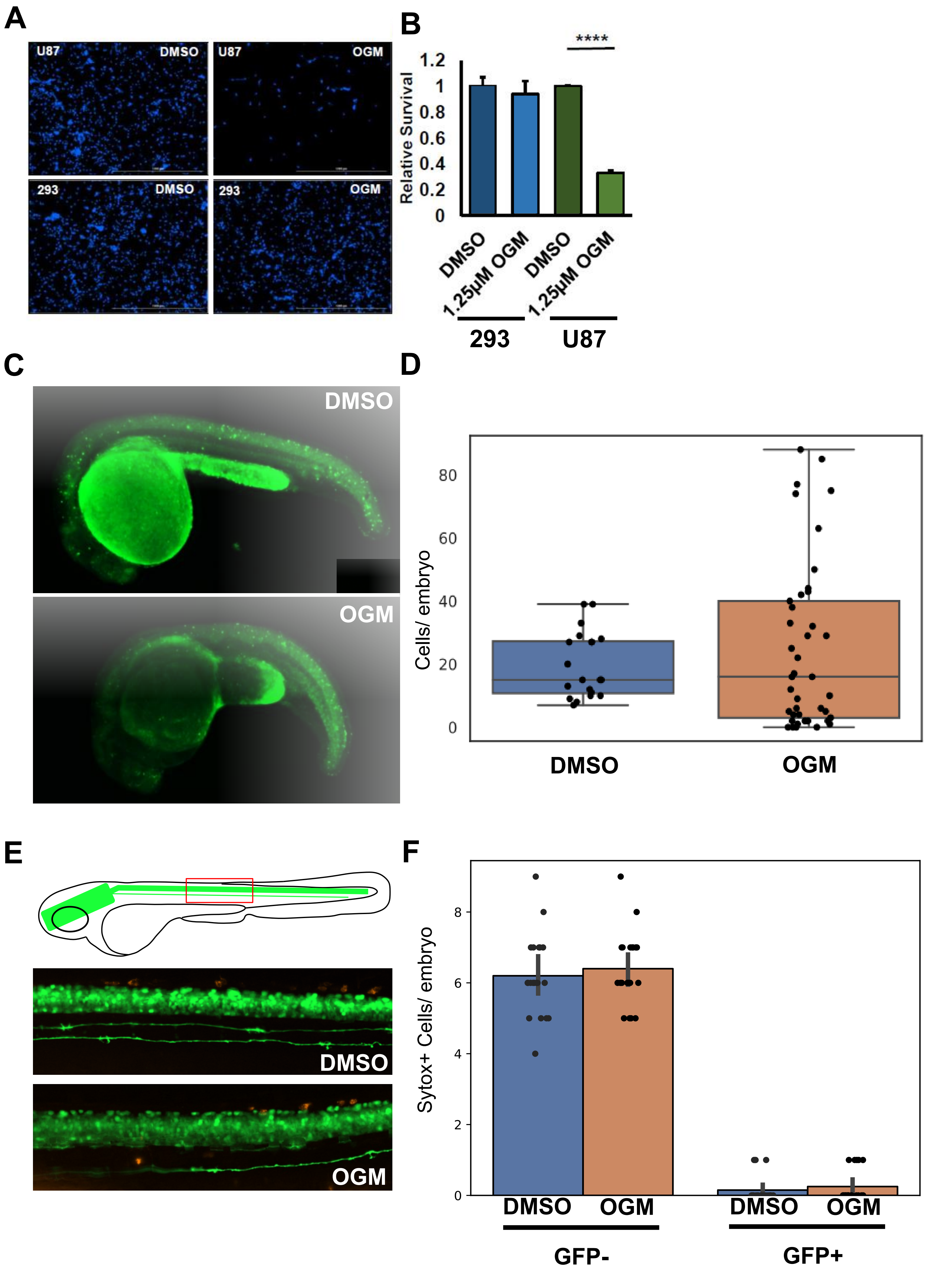
A
U87
DMSO
U87
OGM
293
DMSO
293
OGM
B
****
Relative Survival
1.2
1
0.8
0.6
0.4
0.2
0
DMSO
1.25μM OGM
DMSO
1.25μM OGM
293
U87
C
DMSO
OGM
D
Cells/ embryo
80
60
40
20
0
DMSO
OGM
E
DMSO
OGM
F
Sytox+ Cells/ embryo
8
6
4
2
0
DMSO
OGM
DMSO
OGM
GFP-
GFP+

Figure S10

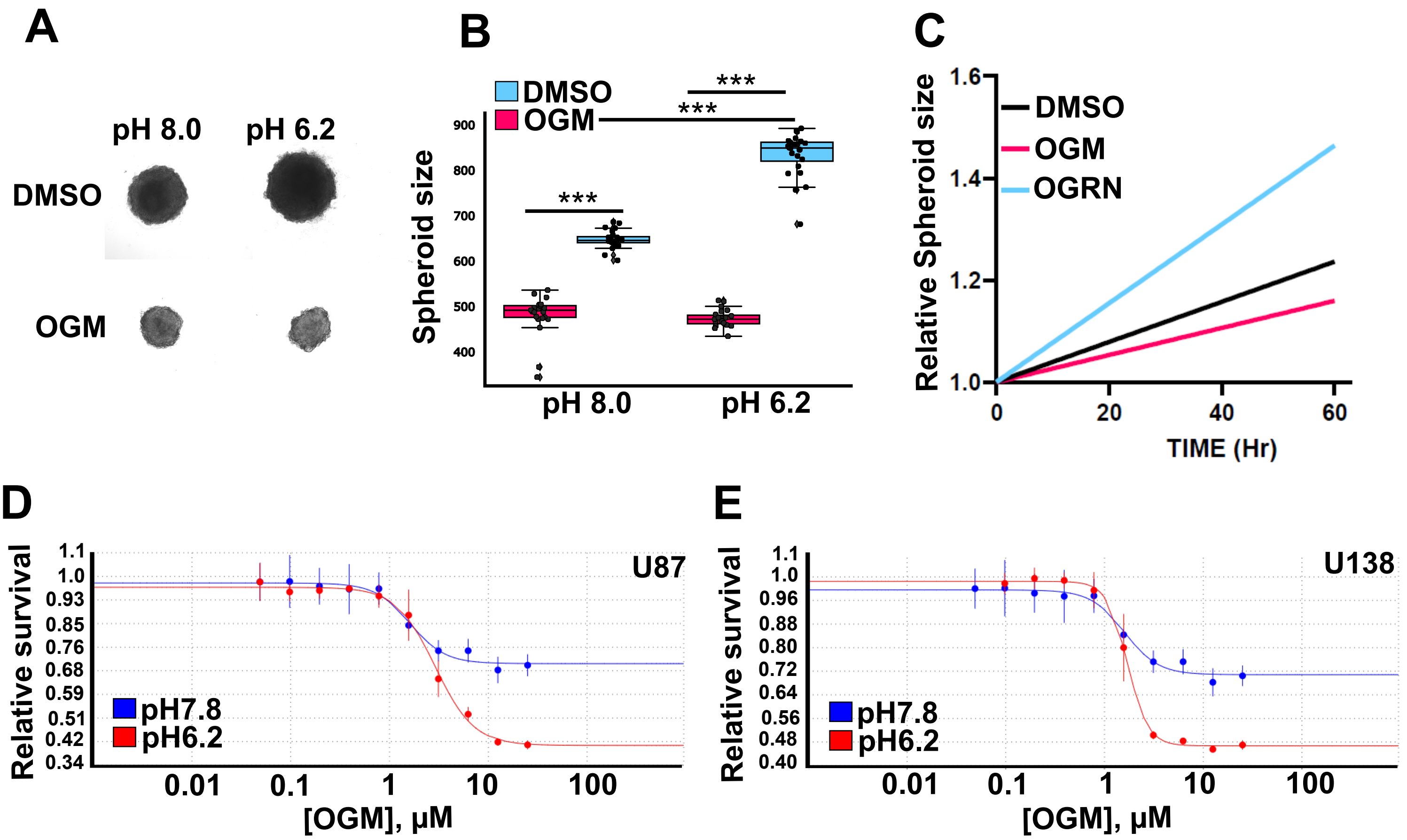

A
pH 8.0
pH 6.2
DMSO
OGM
B
DMSO
OGM
***
***
***
Spheroid size
900
800
700
600
500
400
pH 8.0
pH 6.2
C
Relative Spheroid size
1.6
1.4
1.2
1.0
DMSO
OGM
OGRN
0
20
40
60
TIME (Hr)
D
Relative survival
1.1
1.0
0.93
0.85
0.76
0.68
0.59
0.51
0.42
0.34
U87
pH7.8
pH6.2
0.01
0.1
1
10
100
[OGM], μM
E
Relative survival
1.1
1.0
0.96
0.88
0.80
0.72
0.64
0.56
0.48
0.40
U138
pH7.8
pH6.2
0.01
0.1
1
10
100
[OGM], μM

Figure S11

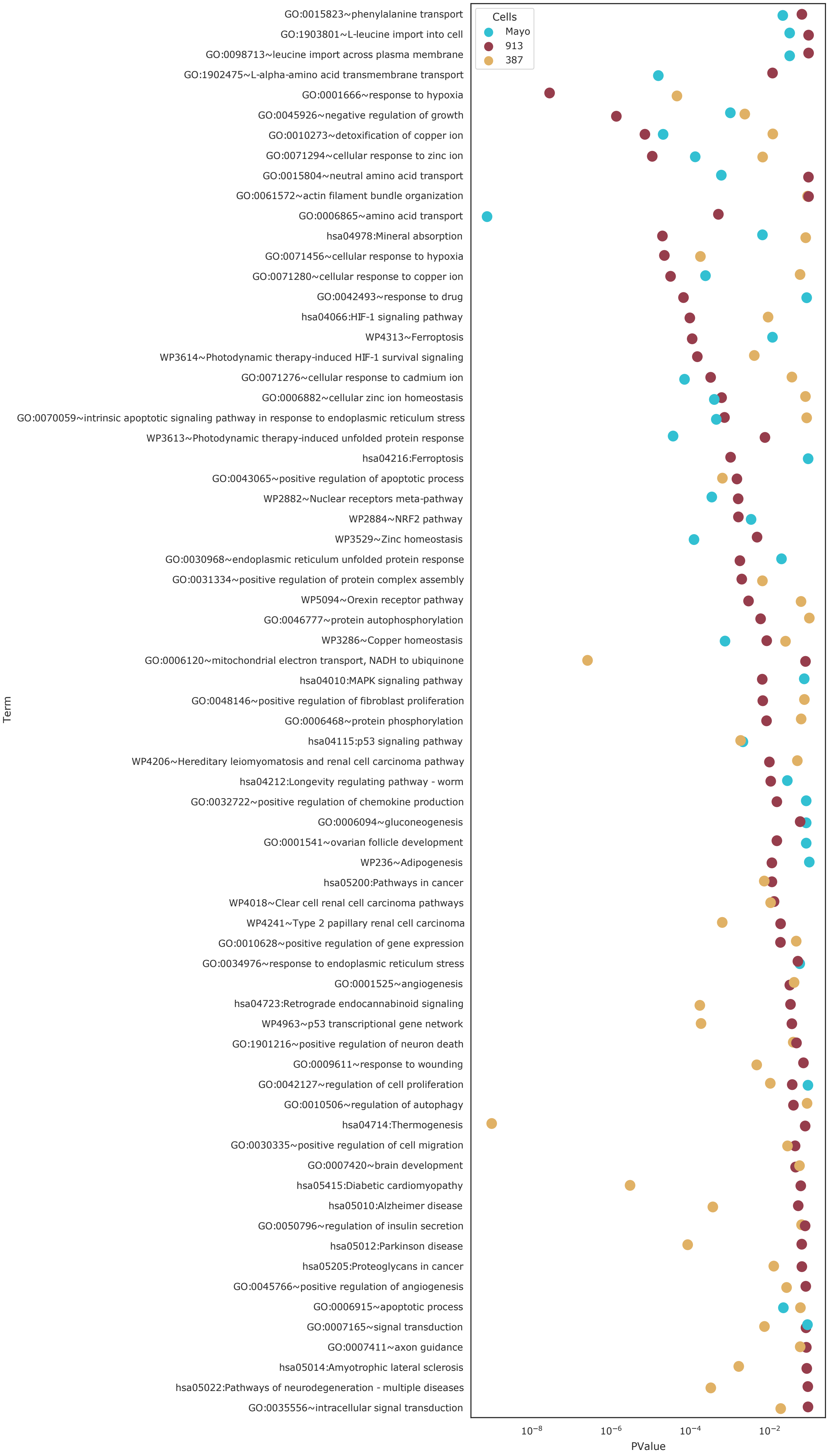
Cells
Mayo
913
387
GO:0015823~phenylalanine transport
GO:1903801~L-leucine import into cell
GO:0098713~leucine import across plasma membrane
GO:1902475~L-alpha-amino acid transmembrane transport
GO:0001666~response to hypoxia
GO:0045926~negative regulation of growth
GO:0010273~detoxification of copper ion
GO:0071294~cellular response to zinc ion
GO:0015804~neutral amino acid transport
GO:0061572~actin filament bundle organization
GO:0006865~amino acid transport
hsa04978:Mineral absorption
GO:0071456~cellular response to hypoxia
GO:0071280~cellular response to copper ion
GO:0042493~response to drug
hsa04066:HIF-1 signaling pathway
WP4313~Ferroptosis
WP3614~Photodynamic therapy-induced HIF-1 survival signaling
GO:0071276~cellular response to cadmium ion
GO:0006882~cellular zinc ion homeostasis
GO:0070059~intrinsic apoptotic signaling pathway in response to endoplasmic reticulum stress
WP3613~Photodynamic therapy-induced unfolded protein response
hsa04216:Ferroptosis
GO:0043065~positive regulation of apoptotic process
WP2882~Nuclear receptors meta-pathway
WP2884~NRF2 pathway
WP3529~Zinc homeostasis
GO:0030968~endoplasmic reticulum unfolded protein response
GO:0031334~positive regulation of protein complex assembly
WP5094~Orexin receptor pathway
GO:0046777~protein autophosphorylation
WP3286~Copper homeostasis
GO:0006120~mitochondrial electron transport, NADH to ubiquinone
hsa04010:MAPK signaling pathway
GO:0048146~positive regulation of fibroblast proliferation
GO:0006468~protein phosphorylation
hsa04115:p53 signaling pathway
WP4206~Hereditary leiomyomatosis and renal cell carcinoma pathway
hsa04212:Longevity regulating pathway - worm
GO:0032722~positive regulation of chemokine production
GO:0006094~gluconeogenesis
GO:0001541~ovarian follicle development
WP236~Adipogenesis
hsa05200:Pathways in cancer
WP4018~Clear cell renal cell carcinoma pathways
WP4241~Type 2 papillary renal cell carcinoma
GO:0010628~positive regulation of gene expression
GO:0034976~response to endoplasmic reticulum stress
GO:0001525~angiogenesis
hsa04723:Retrograde endocannabinoid signaling
WP4963~p53 transcriptional gene network
GO:1901216~positive regulation of neuron death
GO:0009611~response to wounding
GO:0042127~regulation of cell proliferation
GO:0010506~regulation of autophagy
hsa04714:Thermogenesis
GO:0030335~positive regulation of cell migration
GO:0007420~brain development
hsa05415:Diabetic cardiomyopathy
hsa05010:Alzheimer disease
GO:0050796~regulation of insulin secretion
hsa05012:Parkinson disease
hsa05205:Proteoglycans in cancer
GO:0045766~positive regulation of angiogenesis
GO:0006915~apoptotic process
GO:0007165~signal transduction
GO:0007411~axon guidance
hsa05014:Amyotrophic lateral sclerosis
hsa05022:Pathways of neurodegeneration - multiple diseases
GO:0035556~intracellular signal transduction
Term
10^{-8}
10^{-6}
10^{-4}
10^{-2}
PValue

Figure S12

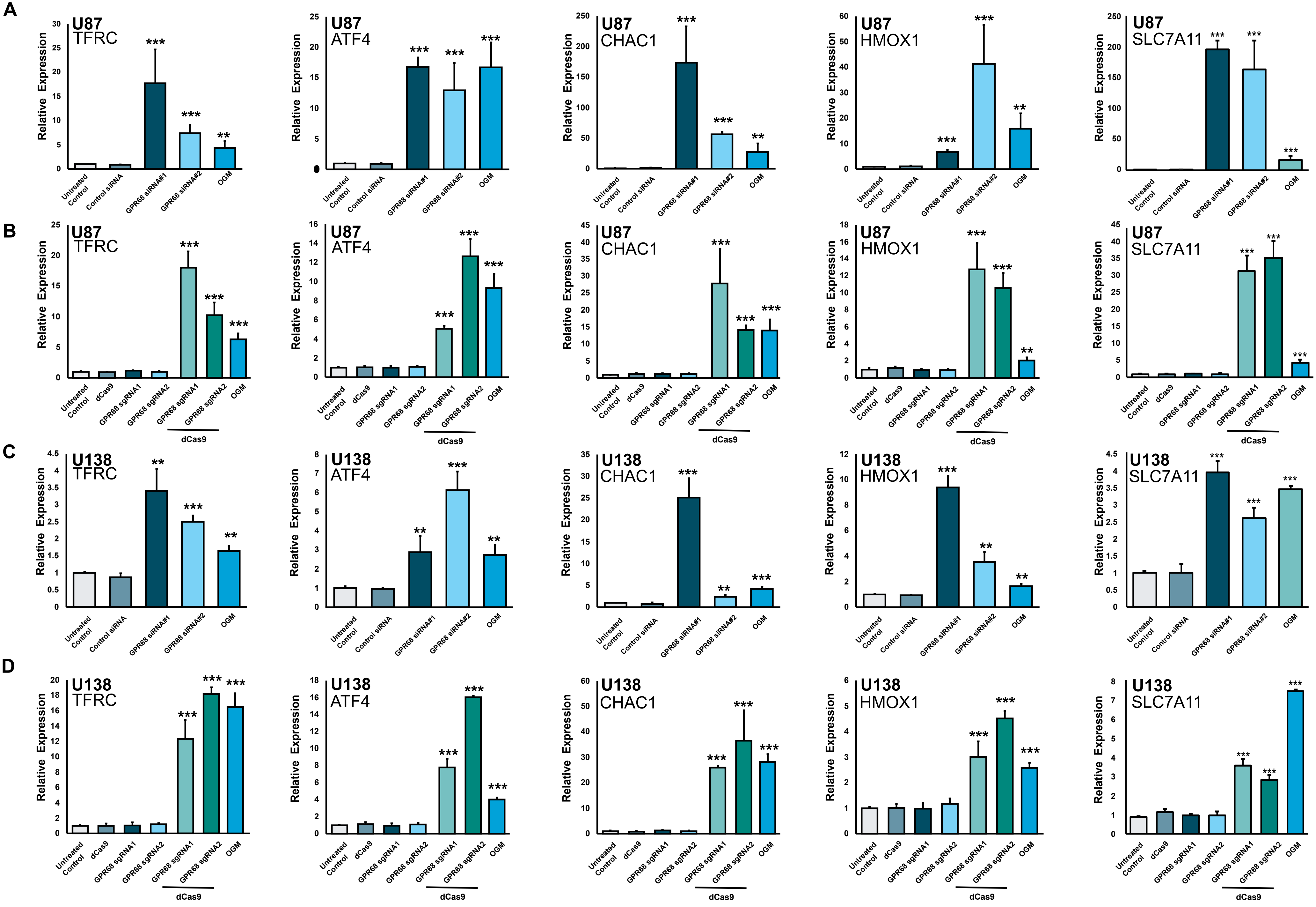
A
U87
TFRC
ATF4
CHAC1
HMOX1
SLC7A11
Relative Expression
Untreated Control
Control siRNA
GPR68 siRNA#1
GPR68 siRNA#2
OGM
B
U87
TFRC
ATF4
CHAC1
HMOX1
SLC7A11
Relative Expression
Untreated Control
dCas9
GPR68 sgRNA1
GPR68 sgRNA2
GPR68 sgRNA1
GPR68 sgRNA2
OGM
dCas9
C
U138
TFRC
ATF4
CHAC1
HMOX1
SLC7A11
Relative Expression
Untreated Control
Control siRNA
GPR68 siRNA#1
GPR68 siRNA#2
OGM
D
U138
TFRC
ATF4
CHAC1
HMOX1
SLC7A11
Relative Expression
Untreated Control
dCas9
GPR68 sgRNA1
GPR68 sgRNA2
GPR68 sgRNA1
GPR68 sgRNA2
OGM
dCas9

Figure S13

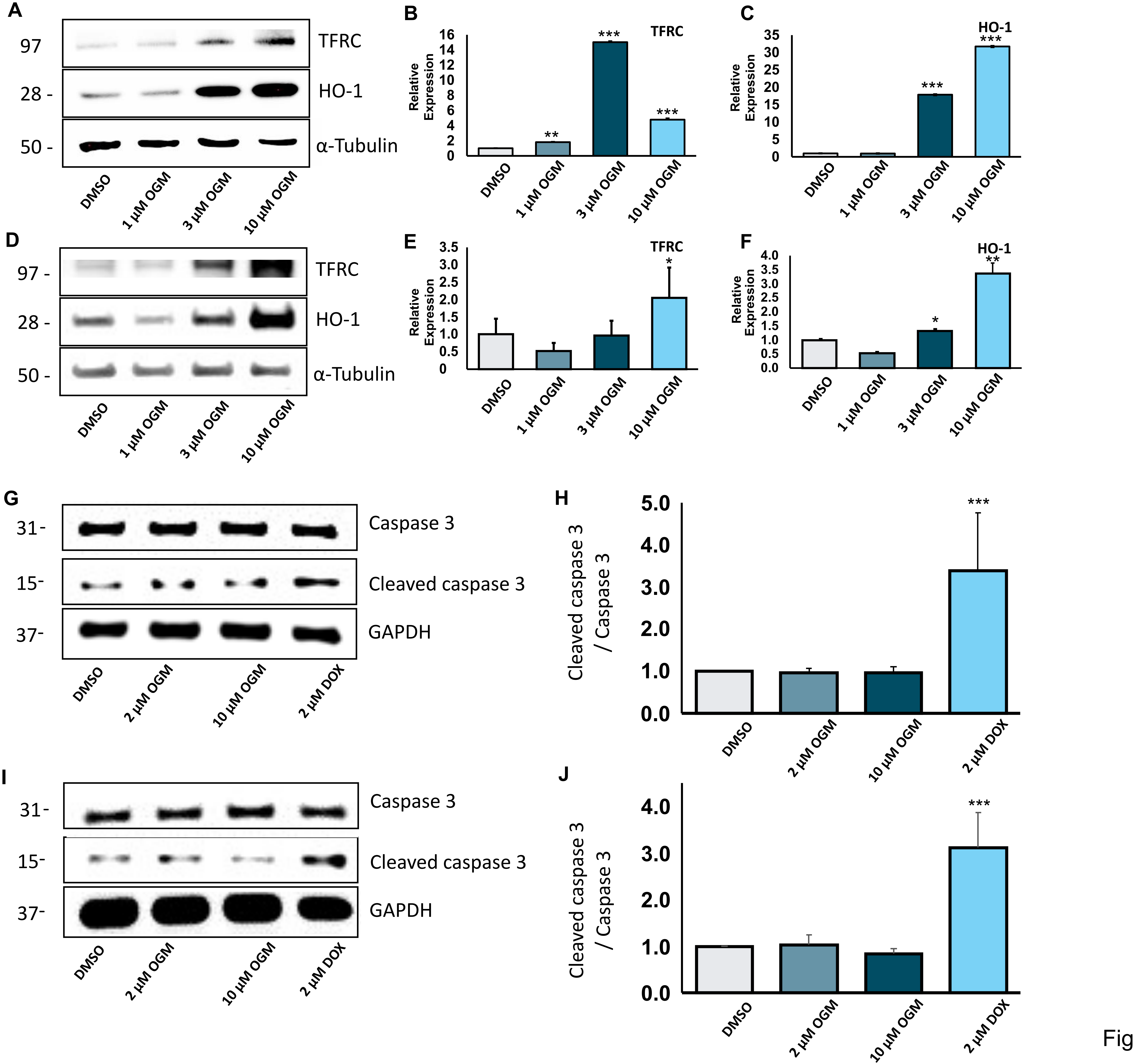
A
97
28 -
50 -
TFRC
HO-1
α-Tubulin
DMSO
1 µM OGM
3 µM OGM
10 µM OGM
B
TFRC
Relative Expression
C
HO-1
Relative Expression
D
97 -
28 -
50 -
TFRC
HO-1
α-Tubulin
E
TFRC
F
HO-1
G
31-
15-
37-
Caspase 3
Cleaved caspase 3
GAPDH
DMSO
2 µM OGM
10 µM OGM
2 µM DOX
H
Cleaved caspase 3 / Caspase 3
I
J

Figure S14

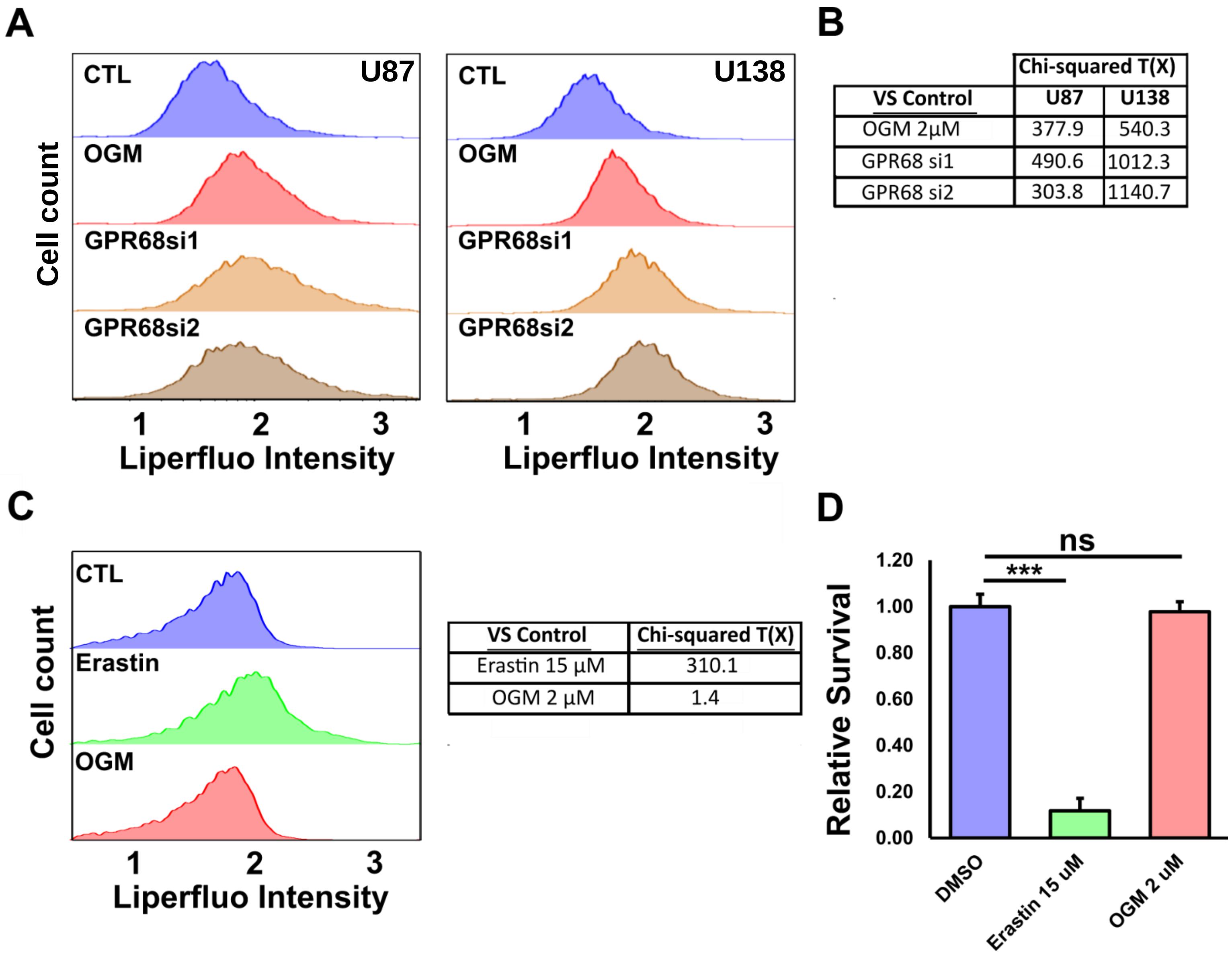

VS Control	Chi-squared T(X) U87	Chi-squared T(X) U138
OGM 2μM	377.9	540.3
GPR68 si1	490.6	1012.3
GPR68 si2	303.8	1140.7

VS Control	Chi-squared T(X)
Erastin 15 μM	310.1
OGM 2 μM	1.4

A

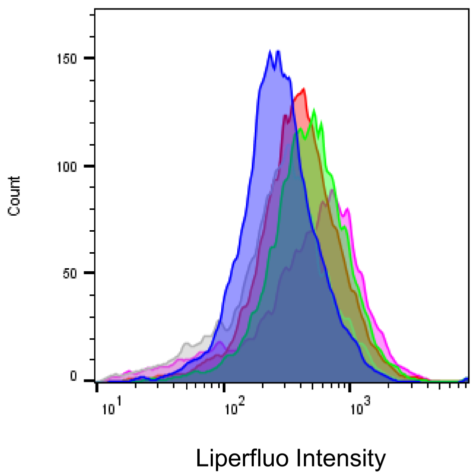

B

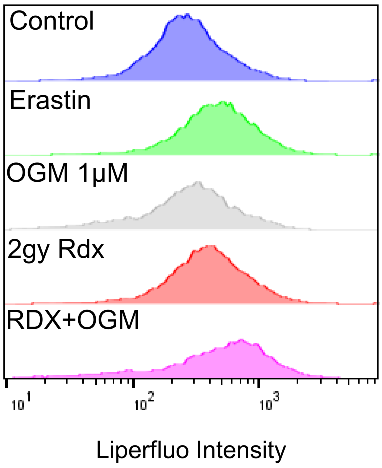

C

CDI=0.06

Stats	Chi T(X)
Stained control Vs. Erastin	305.9
Stained control Vs. OGM	172.8
Stained control Vs. Radiation	60.4
Stained control Vs. Radiation+OGM	373.6
Radiation Vs. Radiation+OGM	218.2
OGM Vs. Radiation+OGM	135.6

D

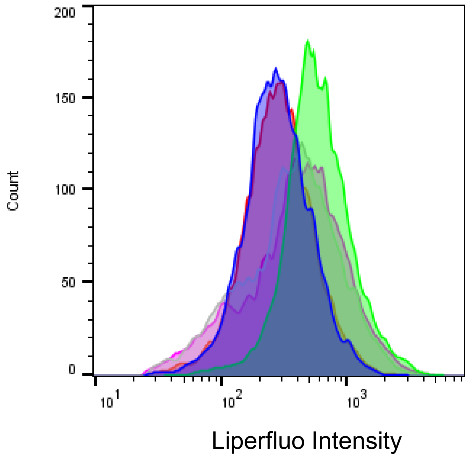

E

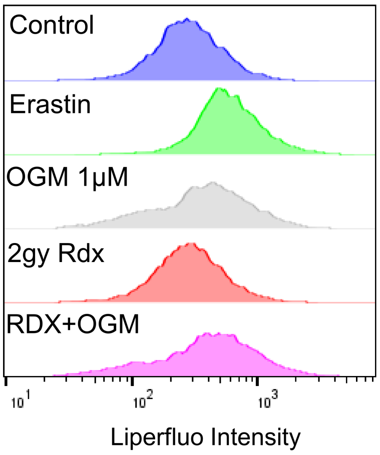

F

CDI=0.006

Stats	Chi T(X)
Stained control Vs. Erastin	742.5
Stained control Vs. OGM	0
Stained control Vs. Radiation	248.2
Stained control Vs. Radiation+OGM	327.6
Radiation Vs. Radiation+OGM	11.1
OGM Vs. Radiation+OGM	287.6

Figure S16

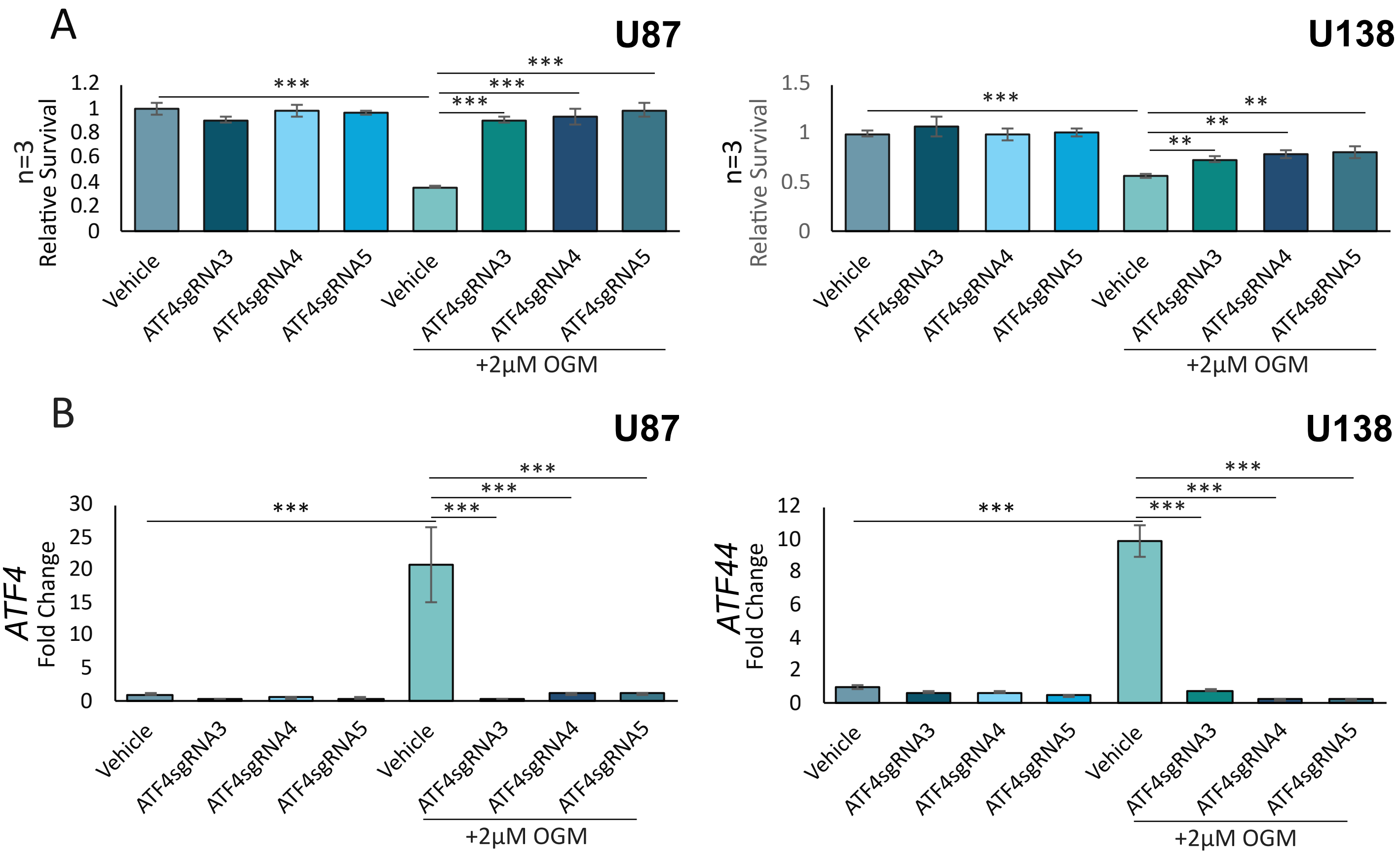
A
U87
n=3
Relative Survival
1.2
1
0.8
0.6
0.4
0.2
0
***
***
***
***
Vehicle
ATF4sgRNA3
ATF4sgRNA4
ATF4sgRNA5
Vehicle
ATF4sgRNA3
ATF4sgRNA4
ATF4sgRNA5
+2µM OGM
U138
n=3
Relative Survival
1.5
1
0.5
0
***
**
**
**
Vehicle
ATF4sgRNA3
ATF4sgRNA4
ATF4sgRNA5
Vehicle
ATF4sgRNA3
ATF4sgRNA4
ATF4sgRNA5
+2µM OGM
B
U87
ATF4
Fold Change
30
25
20
15
10
5
0
***
***
***
***
Vehicle
ATF4sgRNA3
ATF4sgRNA4
ATF4sgRNA5
Vehicle
ATF4sgRNA3
ATF4sgRNA4
ATF4sgRNA5
+2µM OGM
U138
ATF44
Fold Change
12
10
8
6
4
2
0
***
***
***
***
Vehicle
ATF4sgRNA3
ATF4sgRNA4
ATF4sgRNA5
Vehicle
ATF4sgRNA3
ATF4sgRNA4
ATF4sgRNA5
+2µM OGM

Figure S17

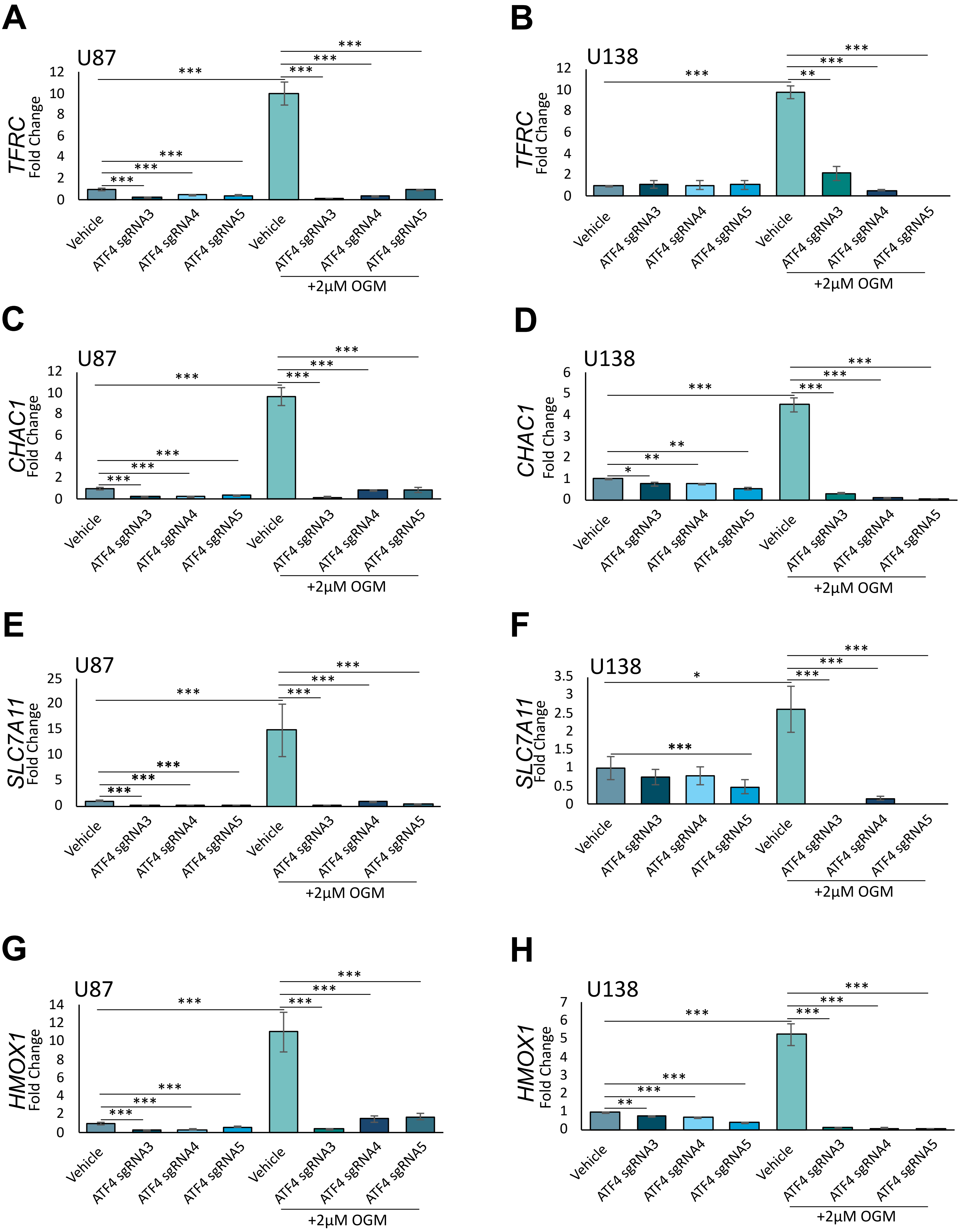

A
U87
TFRC
Fold Change
12
10
8
6
4
2
0
***
Vehicle
ATF4 sgRNA3
ATF4 sgRNA4
ATF4 sgRNA5
+2µM OGM
B
U138
TFRC
Fold Change
**
C
U87
CHAC1
Fold Change
D
U138
CHAC1
Fold Change
6
5
3
1
*
E
U87
SLC7A11
Fold Change
25
20
15
5
F
U138
SLC7A11
Fold Change
3.5
2.5
1.5
0.5
G
U87
HMOX1
Fold Change
14
H
U138
HMOX1
Fold Change
7

Figure S18

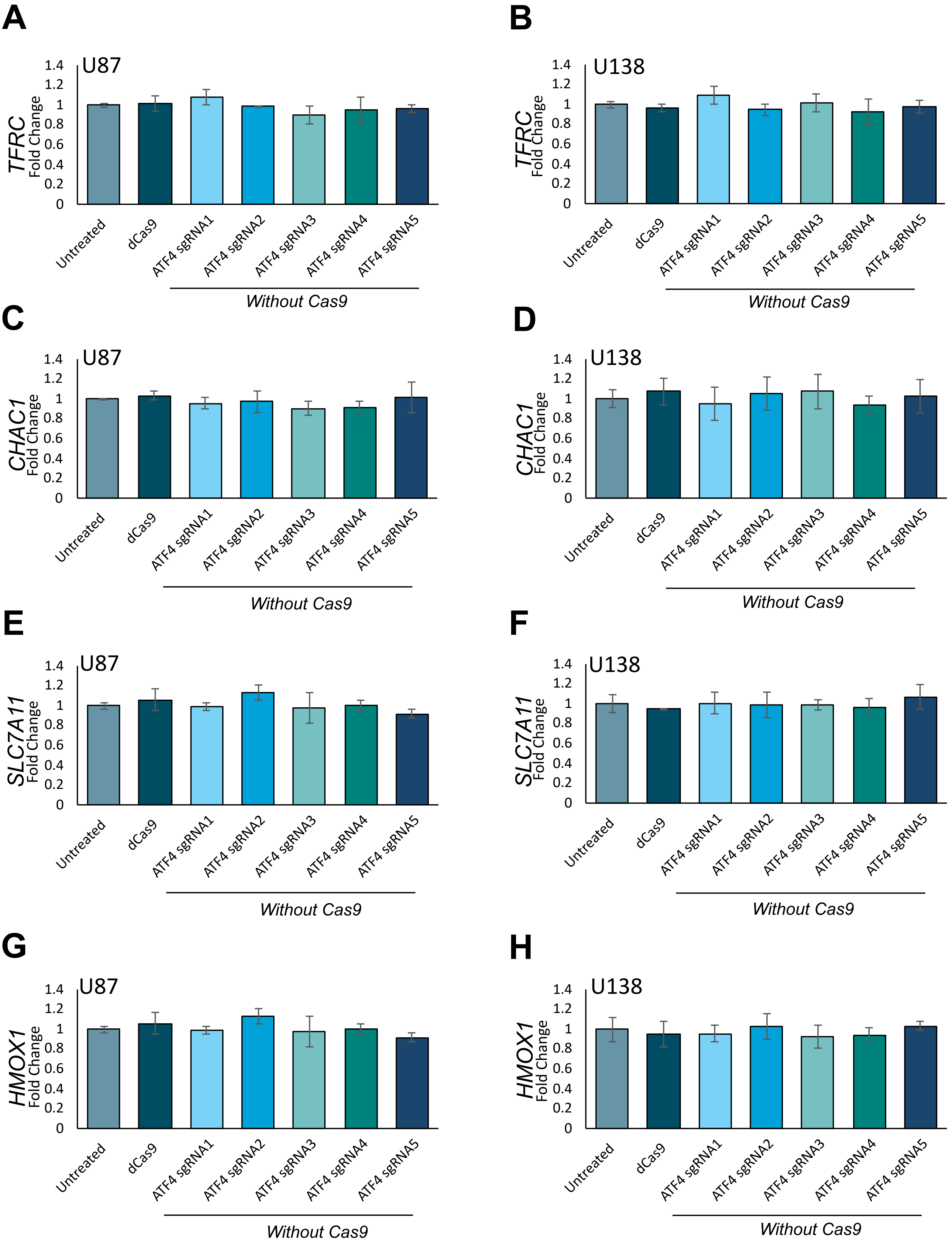
A
U87
TFRC Fold Change
1.4
1.2
1
0.8
0.6
0.4
0.2
0
Untreated
dCas9
ATF4 sgRNA1
ATF4 sgRNA2
ATF4 sgRNA3
ATF4 sgRNA4
ATF4 sgRNA5
Without Cas9
B
U138
TFRC Fold Change
C
U87
CHAC1 Fold Change
D
U138
CHAC1 Fold Change
E
U87
SLC7A11 Fold Change
F
U138
SLC7A11 Fold Change
G
U87
HMOX1 Fold Change
H
U138
HMOX1 Fold Change

Figure S20

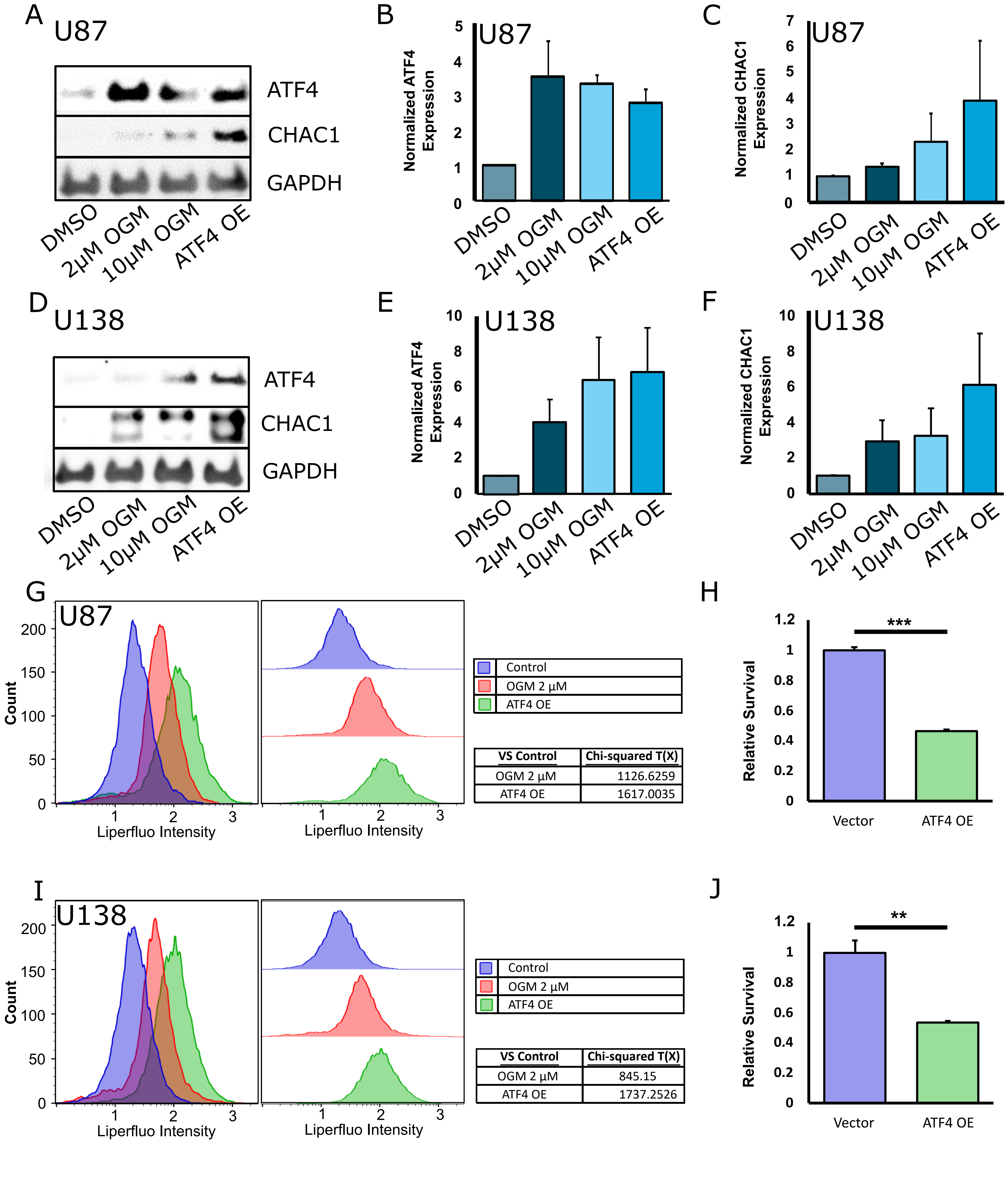
A
U87
ATF4
CHAC1
GAPDH
DMSO
2µM OGM
10µM OGM
ATF4 OE
B
U87
Normalized ATF4 Expression
C
U87
Normalized CHAC1 Expression
D
U138
E
U138
F
U138
G
U87
Count
Liperfluo Intensity
Control
OGM 2 µM
ATF4 OE
VS Control
Chi-squared T(X)
OGM 2 µM 1126.6259
ATF4 OE 1617.0035
H
Relative Survival
***
Vector
ATF4 OE
I
U138
VS Control
Chi-squared T(X)
OGM 2 µM 845.15
ATF4 OE 1737.2526
J
**

Figure S20

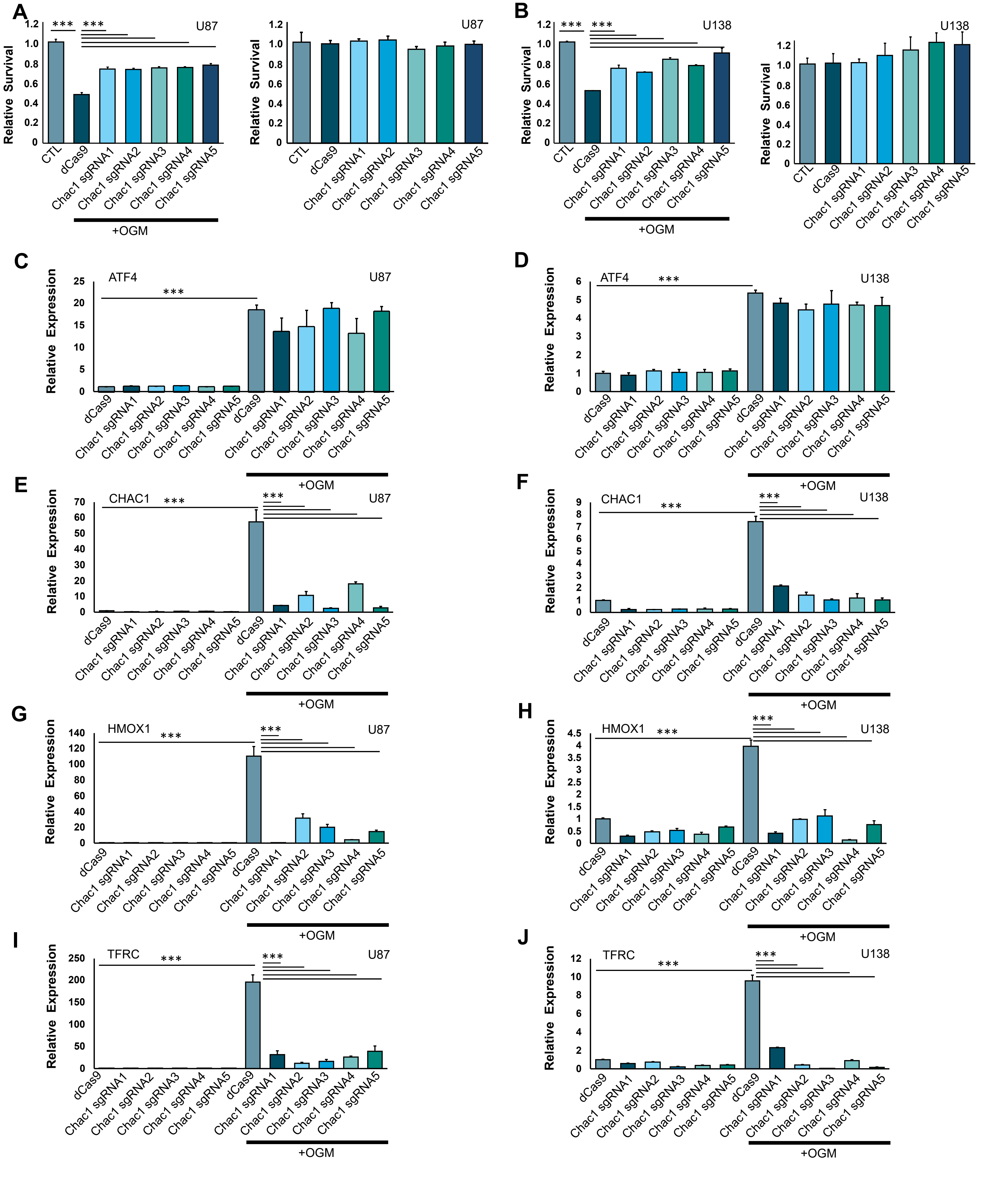
A
U87
Relative Survival
CTL
dCas9
Chac1 sgRNA1
Chac1 sgRNA2
Chac1 sgRNA3
Chac1 sgRNA4
Chac1 sgRNA5
+OGM
B
U138
C
ATF4
Relative Expression
D
E
CHAC1
F
G
HMOX1
H
I
TFRC
J

Figure S21

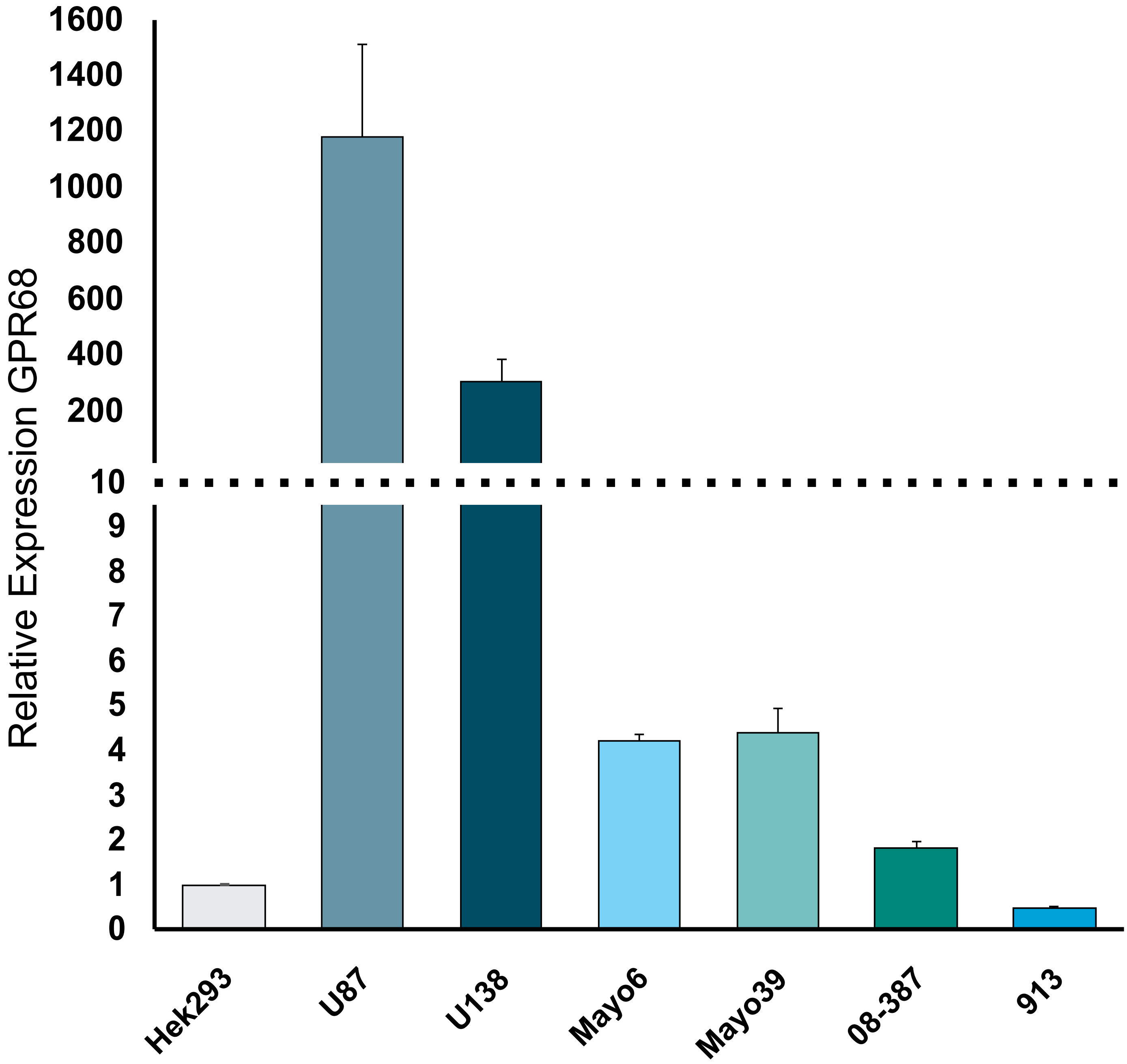
Relative Expression GPR68
1600
1400
1200
1000
800
600
400
200
10
9
8
7
6
5
4
3
2
1
0
Hek293
U87
U138
Mayo6
Mayo39
08-387
913

Figure S22
